# Development and evaluation of novel artemisinin-isatin hybrids with potential anti-leukemic cytotoxicity

**DOI:** 10.3389/fonc.2023.1112369

**Published:** 2023-04-14

**Authors:** Peng Wang, Zhe Zhang, Wei Cao, Xuan Zhang

**Affiliations:** ^1^ Department of Critical Care Unit, Shandong Provincial Hospital Affiliated to Shandong First Medical University, Jinan, Shandong, China; ^2^ Department of Tumor Radiotherapy, Shandong Provincial Hospital Affiliated to Shandong First Medical University, Jinan, Shandong, China; ^3^ Department of Nephrology, The First Affiliated Hospital of Shandong First Medical University & Provincial Qianfoshan Hospital, Shandong Institute of Nephrology, Jinan, Shandong, China; ^4^ Department of Geriatric Respiratory Disease, Shandong Provincial Hospital Affiliated to Shandong First Medical University, Jinan, Shandong, China

**Keywords:** artemisinin, isatin, hybrid molecules, anti-leukemic cytotoxicity, structure-activity relationship

## Abstract

Twenty-one novel ester tethered artemisinin-isatin hybrids were designed, synthesized and screened against human myeloid leukemia cell lines (K562 and K562/ADR), human acute lymphoblastic leukemia cell line (CCRF-CEM) as well as normal human peripheral blood mononuclear cells (PBMCs) for their cytotoxicity by 3-(4,5-dimethylthiazol-2-yl)-2,5-diphenyltetrazolium bromide (MTT) assay. The structure-activity relationships (SARs) were also discussed to facilitate further rational design of more effective candidates. The preliminary results showed that most of the ester tethered artemisinin-isatin hybrids (IC_50_: 0.32-29.35 *µ*M) exhibited promising activity against CCRF-CEM cells, and some of them (IC_50_: 1.23-49.84 *µ*M) were also active against K562 and K562/ADR human myeloid leukemia cell lines. Among them, hybrid 7d (IC_50_: 0.32, 2.67 and 1.23 *µ*M) not only possessed profound activity against the three tested leukemia cell lines and excellent safety and selectivity profiles, but also showed promising pharmacokinetic properties. Accordingly, hybrid 7d could be considered as a potential lead molecule for the development of novel anti-leukemic agents with minimal untoward events to normal human cells.

## 1 Introduction

Acute myeloid leukemia (AML), a common hematological disorder with heterogeneous nature that resulted from blocked myeloid differentiation and an enhanced number of immature myeloid progenitors, is one of the deadliest haematological malignancies and remains a great clinical challenge (1, 2). The survival ratio of patients with AML has greatly improved in recent years which is attributed to the significantly evolution of the therapeutic landscape (3, 4). However, the survival mechanisms utilized by AML cells usually lead to chemoresistance and relapse, and leukemia is projected to be responsible for 470,000 deaths in 2040 if the current trends continue (5, 6). Hence, there is an urgent need for the development of novel anti-leukemic agents.

**Figure 1:**
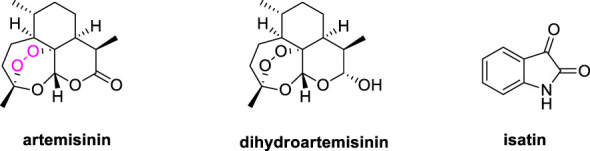
Chemical structures of artemisinin, dihydroartemisinin and isatin.

Artemisinin (ART, **Figure 1**), a sesquiterpene lactone compound owning a peroxyl bridge structure, could significantly increase intracellular reactive oxygen species (ROS) in cancer cells in the presence of ferrous ion (Fe^II^), whereas cancer cells require and uptake a large amount of Fe^II^ to proliferate (7, 8). Furthermore, artemisinin and its derivatives like dihydroartemisinin (DHA) could exert the anticancer effects through promotion of apoptosis, induction of cell cycle arrest and autophagy, as well as inhibition cancer cell invasion and migration (9, 10). Accordingly, artemisinin derivatives possess promising *in vitro* and *in vivo* efficacy against both drug-sensitive and multidrug-resistant cancers including leukaemia.

Isatin derivatives possess diverse mechanisms of action, including binding DNA, generating reactive species that cause oxidative damage, and inhibiting selected proteins, and some of them which are exemplified by Semaxanib, Sunitinib, Intedanib, Nintedanib and Hesperadin are already approved or under clinical evaluations for the treatment of diverse kinds of cancer (11, 12). Additionally, isatin derivatives also possess excellent safety and tolerability profiles (13, 14). Hence, isatin derivatives are useful scaffolds for the development of novel anticancer agents.

Based on the aforementioned facts, combination of artemisinin and isatin moieties may provide novel anticancer candidates. Indeed, the 1,2,3-triazolyl and alkyl tethered artemisinin-isatin hybrids have already demonstrated promising antiproliferative effects against both drug-sensitive and multidrug-resistant cancer cell lines (15, 16). To explore novel anti-leukemic candidates, a series of ester tethered ART-isatin hybrids were designed, synthesis and evaluated for their antiproliferative activity against human myeloid leukemia cell lines (K562 and K562/ADR), human acute lymphoblastic leukemia cell line (CCRF-CEM) as well as cytotoxicity towards normal human peripheral blood mononuclear cells (PBMCs) in this paper. The ester bond is prone to hydrolyze make ester tethered artemisinin-isatin hybrids as potential prodrugs have the potential to overcome poor pharmacokinetic characteristics of artemisinin, inclusive of low water solubility and poor bioavailability.

## 2 Results and discussion

### 2.1 Chemical synthesis

**Scheme 1:**
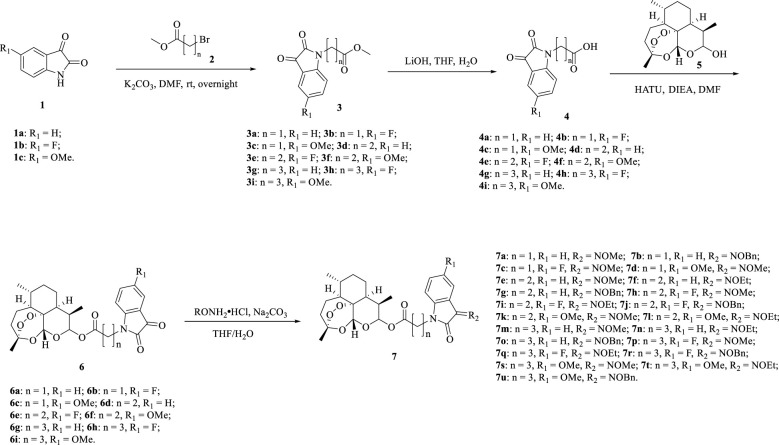
Synthetic route of ester tethered ART-isatin hybrids 7a-u.

The detailed synthetic route of ester tethered ART-isatin hybrids 7a-u was described in **Scheme 1**. Introduction of methyl acetate/propionate/butanoate into *N*-1 position of isatins 1a-c yielded methyl (*N*-1-isatin)acetate/propionate/butanoates 3a-i, which were then hydrolysized to give (*N*-1-isatin)acetic/propionic/butanoic acids 4a-i. Esterification of (*N*-1-isatin)acetic/propionic/butanoic acids 4a-i with DHA (5) provided ART-isatin hybrids 6a-i. The desired ART-isatin hybrids 7a-u were achieved through imidization of ART-isatin hybrids 6a-i with methoxyamine/ethoxyamine/benzyloxyamine hydrochlorides. The structures and yields of ART-isatin hybrids 7a-u were listed in **Table 1**.

**Table 1:** The structures and yields of ester tethered ART-isatin hybrids 7a-u.

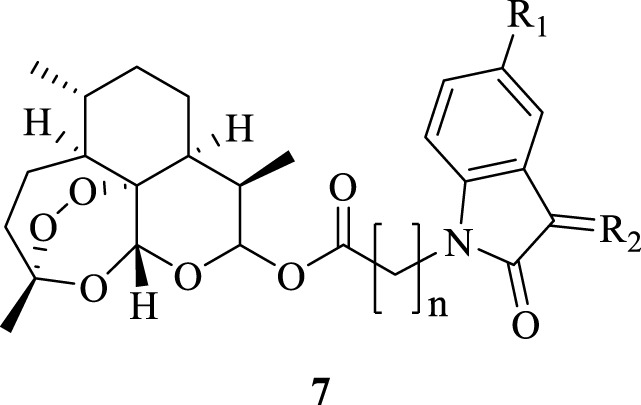	Hybrids	n	R_1_	R_2_	Yield
**7a**	1	H	NOMe	51%
**7b**	1	H	NOBn	67%
**7c**	1	F	NOMe	63%
**7d**	1	OMe	NOMe	49%
**7e**	2	H	NOMe	72%
**7f**	2	H	NOEt	80%
**7g**	2	H	NOBn	95%
**7h**	2	F	NOMe	70%
**7i**	2	F	NOEt	64%
**7j**	2	F	NOBn	89%
**7k**	2	OMe	NOMe	74%
**7l**	2	OMe	NOEt	78%
**7m**	3	H	NOMe	79%
**7n**	3	H	NOEt	66%
**7o**	3	H	NOBn	68%
**7p**	3	F	NOMe	81%
**7q**	3	F	NOEt	78%
**7r**	3	F	NOBn	89%
**7s**	3	OMe	NOMe	88%
**7t**	3	OMe	NOEt	79%
**7u**	3	OMe	NOBn	87%

The desired ester tethered ART-isatin hybrids 7a-u were characterized by high resolution mass spectrometry (HRMS), proton nuclear magnetic resonance (^1^H NMR) and carbon-13 nuclear magnetic resonance spectroscopy (^13^C NMR), and the corresponding analytical spectra were included in the **Supplementary Information** section.

The antiproliferative activity and cytotoxicity of ester tethered ART-isatin hybrids 7a-u against human myeloid leukemia cell lines (K562 CM-0130 purchased from Procell and K562/ADR AW-CELLS-H0187 purchased from AnWei-sci), human acute lymphoblastic leukemia cell line (CCRF-CEM CL-0058 purchased from Procell) as well as cytotoxicity towards normal human peripheral blood mononuclear cells (PBMCs 230470, purchased from Mingzhoubio) were assessed by 3-(4,5-dimethylthiazol-2-yl)-2,5-diphenyltetrazolium bromide (MTT) assay. The antiproliferative activity was expressed by half maximal inhibitory concentration (IC_50_) values, which were presented in **Table 2**.

**Table 2:** The antiproliferative activity of ART-isatin hybrids 7a-u.

Hybrids	Antiproliferative activity (IC_50_: *µ*M)		
	CCRF-CEM	K562	K562/ADR
**7a**	3.83	33.59	49.76
**7b**	>100	>100	>100
**7c**	29.35	>100	>100
**7d**	0.32	2.67	1.23
**7e**	15.17	3.78	22.72
**7f**	9.16	5.70	>100
**7g**	>100	>100	>100
**7h**	1.31	>100	16.25
**7i**	>100	15.93	>100
**7j**	>100	>100	3.83
**7k**	6.74	>100	>100
**7l**	19.36	>100	>100
**7m**	2.78	>100	>100
**7n**	>100	>100	>100
**7o**	>100	>100	>100
**7p**	18.64	49.84	>100
**7q**	12.73	16.02	>100
**7r**	9.21	6.32	>100
**7s**	3.82	>100	>100
**7t**	1.90	>100	>100
**7u**	2.45	>100	>100
**ART**	>100	>100	>100
Adriamycin	0.01	4.89	>100
Vorinostat	0.46	3.83	2.18

From **Table 2**, it can be seen that most of the synthesized hybrids (IC_50_: 0.32-29.35 *µ*M) demonstrated promising activity against CCRF-CEM cells, and some of them (IC_50_: 1.23-49.84 *µ*M) were also active against K562 and K562/ADR human myeloid leukemia cell lines. From the cell images shown in the **Supplementary Material**, no changes on the cell morphology of CCRF-CEM cells were observed after treatment of hybrid 7d compared to before treatment, but cell counts decreased dramatically. For K562 cells, the cells were in a single round shape with bright round, and occasionally a small number of cells were seen in clusters before treatment of hybrid 7d. After treatment, the number of cells decreased significantly, presenting a regular cell shape with bright sides, and most of the cells grew in clusters. The SARs illustrated that (1) the length of ester linkers had remarkably impact on the activity, and acetate and butanoate were generally more favorable to propionate; (2) introduction of electron-donating methoxy into C-5 position of isatin moiety could enhance the activity to some extent; (3) methoxime, ethoxime and benzoxime at C-3 position of isatin influenced the activity, and the relative contribution order was methoxime > ethoxime > benzoxime.

Fifteen hybrids that were active against CCRF-CEM cells were selected for further assessed for their cytotoxicity towards normal human peripheral blood mononuclear cells (PBMCs). From **Table 3**, all of the selected hybrids (IC_50_: >100 *µ*M) were non-toxic towards PBMCs, while the reference adriamycin (IC_50_: 46.9 *µ*M) displayed some toxicity, demonstrating the excellent safety profile of selected hybrids. In addition, the selectivity index (SI: IC_50(PBMCs)_/IC_50(CCRF-CEM)_) values of selected hybrids were >2.54, revealing their excellent selectivity.

**Table 3:** The cytotoxicity, and selectivity index values of selected hybrids.

Hybrids	Cytotoxicity (IC_50_: *µ*M)	SI^a^
**7a**	>100	>26.10
**7c**	>100	>2.54
**7d**	>100	>312.5
**7e**	>100	>6.59
**7f**	>100	>10.91
**7h**	>100	>76.33
**7k**	>100	>14.83
**7l**	>100	>5.16
**7m**	>100	>35.97
**7p**	>100	>5.36
**7q**	>100	>7.85
**7r**	>100	>10.85
**7s**	>100	>26.17
**7t**	>100	>52.63
**7u**	>100	>40.81
Adriamycin	46.9	4690

a SI, Selectivity index, IC_50(PBMCs)_/IC_50(CCRF-CEM)_.

Among them, the representative hybrid 7d (IC_50_: 0.32, 2.67 and 1.23 *µ*M) exhibited eminent activity against CCRF-CEM, K562 and K562/ADR leukemia cell lines, and the activity was superior to that of Vorinostat (IC_50_: 0.46, 3.83 and 2.18 *µ*M) against all the three tested leukemia cell lines and was >81.3 times higher than that of Adriamycin (IC_50_: >100 *µ*M) against K562/ADR cells. The resistance index (RI: IC_50(K562)_/IC_50(K562/ADR)_) value of hybrid 7d was as low as 0.46, revealing its potential to overcome drug resistance. In addition, hybrid 7d (IC_50_: >100 *µ*M) was non-toxic towards PBMCs, and SI value was >312.5, proving its excellent safety and selectivity profiles.

The pharmacokinetic studies indicated that hybrid 7d (30 mg/kg, single intravenous injection) showed the maximum plasma concentration (*C*_max_) of 14.6 *μ*M, area under curve (AUC) of 3,142 n•h/mL, half-life time (*t*_1/2_) of 1.2 h, peak time of 11 min, clearance rates (Cl) of 4.86 L/h/kg, and bioavailability of 44.9% (**Table 4**). The short half-life time may be attributed to the ester linker between DHA and isatin was prone to hydrolyze to release free DHA under the *in vivo* condition. In that case, hybrid 7d could serve as a prodrug.

**Table 4:** Pharmacokinetic properties of hybrid 7d in mice.

Parameter	*C*_max_ (*µ*M)	AUC (ng•h/mL)	*t*_1/2_ (h)	*T*_max_ (min)	Cl (L/h/kg)	*F*(%)
**7d**	14.6	3,142	1.2	11	4.86	44.9

## 3 Conclusion

In conclusion, a series of ester tethered artemisinin-isatin hybrids were designed, synthesis and evaluated for their antiproliferative activity against CCRF-CEM, K562 and K562/ADR leukemia cell lines as well as cytotoxicity towards normal PBMCs cells. A significant part of them were active against CCRF-CEM cells and were non-toxic towards PBMCs cells. In particular, hybrid 7d (IC_50_: 0.32, 2.67 and 1.23 *µ*M) not only possessed profound activity against the three tested leukemia cell lines and excellent safety and selectivity profiles, but also showed promising pharmacokinetic properties in terms of maximum plasma concentration, area under curve, peak time, clearance rates, and bioavailability. The short half-life time may be due to the ester linker between DHA and isatin was prone to hydrolyze to release free DHA under the *in vivo* condition. If that is true, the ester tethered artemisinin-isatin hybrids could serve as prodrugs of DHA which can overcome the pharmacokinetic barriers of artemisinin derivatives since these hybrids possessed promising solubility and bioavailability. Accordingly, hybrid 7d could act as a promising anti-leukemic chemotherapeutic candidate for further evaluations.

## 4 Experimental section

### 4.1 Materials

^1^H NMR and ^13^C NMR spectra were determined on a Varian Mercury-600 spectrometer in CDCl_3_ or acetone-*d*_6_ using tetramethylsilane (TMS) as an internal standard. Electrospray ionization (ESI) mass spectra were obtained on a MDSSCIEXQ-Tap mass spectrometer. Unless otherwise noted, the reagents were obtained from commercial supplier and were used without further purification. CCRF-CEM CL-0058 and K562 CM-0130 leukemia cell lines were purchased from Procell, while K562/ADR AW-CELLS-H0187 leukemia cell line was purchased from AnWei-sci.

### 4.2 Synthesis

The mixture of isatins 1a-c (50 mmol) and potassium carbonate (K_2_CO_3_, 150 mmol) in *N*,*N*-dimethylformamide (DMF, 50 mL) was stirred at 30°C for 1 h, and then methyl 2-bromoacetate/3-bromopropionate/4-bromobutanoate (70 mmol) was added. The mixture was stirred at 30°C for 12 h, and then filtered. The filtrate was concentrated under reduced pressure, and the residue was purified by silica gel chromatography eluted with petroleum ether (PE) to PE: ethyl acetate (EA) = 2: 1 to generate methyl (*N*-1-isatin)acetate/propionate/butanoates 3a-i. The mixture of methyl (*N*-1-isatin)acetate/propionate/butanoates 3a-i (50 mmol) and lithium hydroxide (LiOH,100 mmol) in a mixture of tetrahydrofuran (THF, 100 mL) and H_2_O (50 mL) was stirred at 30°C for 12 h, and then the pH was adjusted to 4.0 by 1 M hydrochloric acid (HCl). The precipitate was collected and washed with H_2_O (100 mL), and the solid was dried under reduced pressure to provide (*N*-1-isatin)acetic/propionic/butanoic acids 4a-i. The mixture of 4(*N*-1-isatin)acetic/propionic/butanoic acids 4a-i (22 mmol), DHA (5, 20 mmol), 2-(7-azabenzotriazol-1-yl)-*N*,*N*,*N*’,*N*’-tetramethyluronium hexafluorophosphate (HATU, 25 mmol) and diisopropylethylamine (DIEA, 10 mL) in DMF (100 mL) was stirred at 30°C for 12 h, and then concentrated *in vacuo*. The residue was purified by silica gel chromatography eluted with PE to PE: EA = 1: 1 to give ART-isatin hybrids 6a-i. To a solution of ART-isatin hybrids 6a-i (1.0 mmol) and methoxyamine/ethoxyamine/benzyloxyamine hydrochlorides (2.0 mmol) in a mixture of THF (20 mL) and H_2_O (10 mL), sodium carbonate (Na_2_CO_3_, 5.0 mmol) was added. The mixture was stirred at 40°C for 12 h, and then cooled to room temperature. The mixture was extracted with dichloromethane (DCM, 30 mL × 3). The combined organic layers were washed with H_2_O (40 mL) and brine (40 mL) in sequence, dried over anhydrous sodium sulfate (Na_2_SO_4_), filtered and concentrated under reduced pressure. The residue was purified by silica gel chromatography eluted with PE to PE: EA = 1: 1 to give the desired ART-isatin hybrids 7a-u.

#### 4.2.1 (3*R*,5a*S*,6*R*,8a*S*,9*R*,12*R*,12a*R*)-3,6,9-trimethyldecahydro-12*H*-3,12-epoxy[1,2]dioxepino[4,3-*i*]isochromen-10-yl 2-(3-(methoxyimino)-2-oxoindolin-1-yl)acetate (7a)

^1^H NMR (600 MHz, CDCl_3_) δ 0.86-1.03 (m, 7H), 1.27-1.39 (m, 3H), 1.43-1.54 (m, 4H), 1.62-1.66 (m, 1H), 1.71-1.74 (m, 1H), 1.77-1.80 (m, 1H), 1.89-1.92 (m, 1H), 2.03-2.07 (m, 1H), 2.37-2.42 (m, 1H), 2.57-2.61 (m, 1H), 4.32 (s, 3H, NOMe), 4.50 (d, *J* = 12.0 Hz, 1H), 4.72 (d, *J* = 12.0 Hz, 1H), 5.44 (s, 1H), 5.82 (d, *J* = 4.0 Hz, 1H), 6.76 (d, *J* = 4.0 Hz, 1H), 7.10 (t, *J* = 4.0 Hz, 1H), 7.38 (t, *J* = 4.0 Hz, 1H), 8.00 (d, *J* = 4.0 Hz, 1H). ^13^C NMR (150 MHz, CDCl_3_) 164.41, 161.66, 141.29, 141.11, 130.82, 126.08, 121.48, 113.93, 106.93, 102.66, 91.41, 89.63, 78.15, 62.97, 49.38, 43.28, 39.24, 36.34, 34.27, 32.12, 29.89, 23.97, 22.66, 20.04, 18.27, 10.19. HRMS-ESI: m/z Calcd for C_26_H_32_N_2_O_8_Na [M+Na]^+^: 523.2051; Found: 523.2006.

#### 4.2.2 (3*R*,5a*S*,6*R*,8a*S*,9*R*,12*R*,12a*R*)-3,6,9-trimethyldecahydro-12*H*-3,12-epoxy[1,2]dioxepino[4,3-*i*]isochromen-10-yl 2-(3-[(benzyloxy)imino)-2-oxoindolin-1-yl]acetate (7b)

^1^H NMR (600 MHz, CDCl_3_) δ 0.87-1.03 (m, 7H), 1.29-1.39 (m, 3H), 1.44-1.53 (m, 4H), 1.64-1.68 (m, 1H), 1.72-1.80 (m, 2H), 1.90-1.92 (m, 1H), 2.04-2.08 (m, 1H), 2.37-2.43 (m, 1H), 2.60-2.62 (m, 1H), 4.50 (d, *J* = 12.0 Hz, 2H), 4.72 (d, *J* = 12.0 Hz, 2H), 5.45 (s, 1H), 5.68 (s, 2H), 5.84 (d, *J* = 4.0 Hz, 1H), 6.76 (d, *J* = 4.0 Hz, 1H), 7.06 (t, *J* = 4.0 Hz, 1H), 7.36-7.43 (m, 4H), 7.46-7.48 (m, 2H), 7.98 (d, *J* = 4.0 Hz, 1H). ^13^C NMR (150 MHz, CDCl_3_) 166.33, 163.80, 143.64, 143.02, 136.14, 132.67, 128.64, 128.51, 128.48, 128.16, 123.46, 116.89, 108.82, 104.38, 93.33, 91.61, 80.06, 79.82, 51.49, 45.19, 41.16, 37.25, 36.18, 34.03, 31.81, 26.88, 24.67, 21.96, 20.18, 12.10. HRMS-ESI: m/z Calcd for C_28_H_36_N_2_O_8_Na [M+Na]^+^: 599.2364; Found: 599.2326.

#### 4.2.3 (3*R*,5a*S*,6*R*,8a*S*,9*R*,12*R*,12a*R*)-3,6,9-trimethyldecahydro-12*H*-3,12-epoxy[1,2]dioxepino[4,3-*i*]isochromen-10-yl 2-(5-fluoro-3-(methoxyimino)-2-oxoindolin-1-yl)acetate (7c)

^1^H NMR (600 MHz, CDCl_3_) δ 0.72-0.92 (m, 7H), 1.09-1.42 (m, 7H), 1.47-1.64 (m, 3H), 1.74-1.78 (m, 1H), 1.89-1.92 (m, 1H), 2.14-2.19 (m, 1H), 2.31-2.34 (m, 1H), 4.16 (s, 3H, NOMe), 4.56-4.64 (m, 2H), 5.41 (s, 1H), 5.66 (d, *J* = 8.0 Hz, 1H), 6.96-6.99 (m, 1H), 7.12-7.16 (m, 1H), 7.62-7.64 (m, 1H). HRMS-ESI: m/z Calcd for C_26_H_31_FN_2_O_8_Na [M+Na]^+^: 541.1957; Found: 541.1916.

#### 4.2.4 (3*R*,5a*S*,6*R*,8a*S*,9*R*,12*R*,12a*R*)-3,6,9-trimethyldecahydro-12*H*-3,12-epoxy[1,2]dioxepino[4,3-*i*]isochromen-10-yl 2-(5-fluoro-3-(methoxyimino)-2-oxoindolin-1-yl)acetate (7d)

^1^H NMR (600 MHz, CDCl_3_) δ 0.78-0.96 (m, 7H), 1.19-1.41 (m, 7H), 1.54-1.57 (m, 1H), 1.63-1.71 (m, 2H), 1.81-1.83 (m, 1H), 1.94-1.98 (m, 1H), 2.28-2.34 (m, 1H), 2.49-2.53 (m, 1H), 3.74 (s, 3H, OMe), 4.25 (s, 3H, NOMe), 4.36 (d, *J* = 12.0 Hz, 1H), 4.62 (d, *J* = 12.0 Hz, 1H), 5.36 (s, 1H), 5.74 (d, *J* = 8.0 Hz, 1H), 6.60 (d, *J* = 4.0 Hz, 1H), 6.84 (dd, *J* = 4.0, 2.0 Hz, 1H), 7.52 (d, *J* = 2.0 Hz, 1H). ^13^C NMR (150 MHz, CDCl_3_) 166.39, 163.41, 156.10, 143.48, 136.79, 117.69, 116.38, 114.42, 109.33, 104.86, 93.27, 91.60, 80.06, 64.94, 56.00, 51.40, 45.19, 41.25, 37.25, 36.18, 34.03, 31.80, 26.89, 24.67, 21.96, 20.18, 12.11. HRMS-ESI: m/z Calcd for C_26_H_31_FN_2_O_8_Na [M+Na]^+^: 541.1957; Found: 541.1916.

#### 4.2.5 (3*R*,5a*S*,6*R*,8a*S*,9*R*,12*R*,12a*R*)-3,6,9-trimethyldecahydro-12*H*-3,12-epoxy[1,2]dioxepino[4,3-*i*]isochromen-10-yl 3-(3-(methoxyimino)-2-oxoindolin-1-yl)propanoate (7e)

^1^H NMR (600 MHz, CDCl_3_) δ 0.70-0.96 (m, 7H), 1.19-1.30 (m, 3H), 1.36-1.45 (m, 4H), 1.52-1.55 (m, 1H), 1.63-1.69 (m, 2H), 1.80-1.83 (m, 1H), 1.94-1.98 (m, 1H), 2.27-2.33 (m, 1H), 2.46-2.48 (m, 1H), 2.76 (t, *J* = 4.0 Hz, 1H), 3.92-4.06 (m, 2H), 4.22 (s, 3H, NOMe), 5.36 (s, 1H), 5.70 (d, *J* = 4.0 Hz, 1H), 6.90 (d, *J* = 4.0 Hz, 1H), 6.98 (t, *J* = 4.0 Hz, 1H), 7.34 (t, *J* = 4.0 Hz, 1H), 7.88 (d, *J* = 4.0 Hz, 1H). ^13^C NMR (150 MHz, CDCl_3_) 169.99, 163.66, 143.41, 143.27, 132.89, 127.99, 123.05, 116.84, 108.94, 104.80, 92.34, 91.61, 80.07, 64.80, 61.83, 46.20, 37.28, 36.20, 35.68, 34.05, 32.25, 31.82, 25.94, 24.67, 21.97, 20.20, 12.08. HRMS-ESI: m/z Calcd for C_27_H_34_N_2_O_8_Na [M+Na]^+^: 537.2207; Found: 537.2170.

#### 4.2.6 (3*R*,5a*S*,6*R*,8a*S*,9*R*,12*R*,12a*R*)-3,6,9-trimethyldecahydro-12*H*-3,12-epoxy[1,2]dioxepino[4,3-*i*]isochromen-10-yl 3-(3-(ethoxyimino)-2-oxoindolin-1-yl)propanoate (7f)

^1^H NMR (600 MHz, CDCl_3_) δ 0.72-0.96 (m, 7H), 1.20-1.28 (m, 3H), 1.36-1.41 (m, 7H), 1.51-1.69 (m, 3H), 1.80-1.83 (m, 1H), 1.94-1.98 (m, 1H), 2.27-2.33 (m, 1H), 2.46-2.48 (m, 1H), 2.76 (t, *J* = 4.0 Hz, 2H), 3.93-4.04 (m, 2H), 4.48 (q, *J* = 4.0 Hz, 2H), 5.36 (s, 1H), 5.70 (d, *J* = 4.0 Hz, 1H), 6.88 (d, *J* = 8.0 Hz, 1H), 6.98-7.01 (m, 1H), 7.32 (d, *J* = 8.0 Hz, 1H), 7.89-7.91 (m, 1H). ^13^C NMR (150 MHz, CDCl_3_) 170.03, 163.71, 143.14, 132.41, 132.36, 127.90, 123.08, 123.01, 116.98, 108.87, 104.61, 92.33, 91.62, 80.08, 73.11, 73.06, 61.61, 45.21, 37.27, 36.20, 36.05, 34.70, 34.06, 32.28, 31.89, 31.62, 26.94, 24.67, 21.97, 20.20, 14.72, 12.08. HRMS-ESI: m/z Calcd for C_28_H_36_N_2_O_8_Na [M+Na]^+^: 551.2364; Found: 551.2344.

#### 4.2.7 (3*R*,5a*S*,6*R*,8a*S*,9*R*,12*R*,12a*R*)-3,6,9-trimethyldecahydro-12*H*-3,12-epoxy[1,2]dioxepino[4,3-*i*]isochromen-10-yl 3-(3-[(benzyloxy)imino)-2-oxoindolin-1-yl]propanoate (7g)

^1^H NMR (600 MHz, CDCl_3_) δ 0.71-0.95 (m, 7H), 1.20-1.30 (m, 3H), 1.36-1.41 (m, 7H), 1.52-1.54 (m, 1H), 1.63-1.67 (m, 2H), 1.81-1.83 (m, 1H), 1.94-1.98 (m, 1H), 2.27-2.33 (m, 1H), 2.46-2.48 (m, 1H), 2.76 (t, *J* = 4.0 Hz, 2H), 3.92-4.04 (m, 2H), 5.36 (s, 1H), 5.46 (s, 1H), 5.70 (d, *J* = 4.0 Hz, 1H), 6.88 (d, *J* = 8.0 Hz, 1H), 6.96 (t, *J* = 8.0 Hz, 1H), 7.28-7.38 (m, 6H), 7.86 (d, *J* = 8.0 Hz, 1H). ^13^C NMR (150 MHz, CDCl_3_) 170.00, 163.67, 143.79, 143.29, 136.23, 132.64, 128.62, 128.47, 128.44, 128.16, 123.11, 116.90, 108.91, 104.61, 92.34, 91.82, 80.08, 79.41, 61.61, 46.21, 37.28, 36.21, 35.68, 34.07, 32.25, 31.63, 26.96, 24.38, 21.98, 20.21, 12.09. HRMS-ESI: m/z Calcd for C_33_H_38_N_2_O_8_Na [M+Na]^+^: 613.2520; Found: 613.2462.

#### 4.2.8 (3*R*,5a*S*,6*R*,8a*S*,9*R*,12*R*,12a*R*)-3,6,9-trimethyldecahydro-12*H*-3,12-epoxy[1,2]dioxepino[4,3-*i*]isochromen-10-yl 3-(5-fluoro-3-(methoxyimino)-2-oxoindolin-1-yl)propanoate (7h)

^1^H NMR (600 MHz, CDCl_3_) δ 0.72-0.96 (m, 7H), 1.20-1.41 (m, 8H), 1.53-1.56 (m, 1H), 1.63-1.70 (m, 2H), 1.81-1.83 (m, 1H), 1.94-1.98 (m, 1H), 2.28-2.33 (m, 1H), 2.42-2.48 (m, 1H), 3.74 (t, *J* = 4.0 Hz, 1H), 3.92-4.03 (m, 2H), 4.24 (s, 3H, NOMe), 5.36 (s, 1H), 5.68 (d, *J* = 4.0 Hz, 1H), 6.88 (dd, *J* = 4.0, 2.0 Hz, 1H), 7.06 (td, *J* = 8.0, 2.0 Hz, 1H), 7.62 (dd, *J* = 8.0, 2.0 Hz, 1H). ^13^C NMR (150 MHz, CDCl_3_) 170.04, 163.37, 159.83, 158.03, 143.03, 139.36, 118.91, 118.75, 116.30, 116.24, 115.61, 115.34, 109.80, 109.75, 104.61, 92.38, 91.61, 80.06, 65.06, 51.62, 46.19, 37.28, 36.19, 35.84, 34.06, 32.35, 31.61, 25.99, 24.57, 21.97, 20.20, 12.07. HRMS-ESI: m/z Calcd for C_27_H_33_FN_2_O_8_Na [M+Na]^+^: 555.2113; Found: 555.2077.

#### 4.2.9 (3*R*,5a*S*,6*R*,8a*S*,9*R*,12*R*,12a*R*)-3,6,9-trimethyldecahydro-12*H*-3,12-epoxy[1,2]dioxepino[4,3-*i*]isochromen-10-yl 3-(3-(ethoxyimino)-5-fluoro-2-oxoindolin-1-yl)propanoate (7i)

^1^H NMR (600 MHz, CDCl_3_) δ 0.72-0.95 (m, 7H), 1.20-1.29 (m, 3H), 1.36-1.41 (m, 7H), 1.53-1.56 (m, 1H), 1.63-1.66 (m, 2H), 1.81-1.83 (m, 1H), 1.94-1.98 (m, 1H), 2.28-2.31 (m, 1H), 2.46-2.47 (m, 1H), 2.75 (t, *J* = 4.0 Hz, 2H), 3.93-4.02 (m, 2H), 4.50 (q, *J* = 4.0 Hz, 2H), 5.36 (s, 1H), 5.68 (d, *J* = 8.0 Hz, 1H), 6.88 (dd, *J* = 8.0, 4.0 Hz, 1H), 7.06 (td, *J* = 8.0, 2.0 Hz, 1H), 7.64 (dd, *J* = 4.0, 2.0 Hz, 1H). ^13^C NMR (150 MHz, CDCl_3_) 170.06, 163.51, 159.63, 158.03, 142.89, 139.24, 118.71, 118.66, 116.42, 116.37, 115.41, 115.23, 109.71, 109.66, 104.62, 92.38, 91.61, 80.06, 73.42, 61.83, 46.20, 37.28, 36.19, 35.81, 34.06, 32.37, 31.61, 25.93, 24.67, 21.97, 20.20, 14.71, 12.07. HRMS-ESI: m/z Calcd for C_27_H_33_FN_2_O_8_Na [M+Na]^+^: 569.2270; Found: 569.2240.

#### 4.2.10 (3*R*,5a*S*,6*R*,8a*S*,9*R*,12*R*,12a*R*)-3,6,9-trimethyldecahydro-12*H*-3,12-epoxy[1,2]dioxepino[4,3-*i*]isochromen-10-yl 3-(3-[(benzyloxy)imino)-5-fluoro-2-oxoindolin-1-yl]propanoate (7j)

^1^H NMR (600 MHz, CDCl_3_) δ 0.71-0.97 (m, 7H), 1.20-1.29 (m, 3H), 1.36-1.42 (m, 4H), 1.53-1.56 (m, 1H), 1.63-1.67 (m, 2H), 1.81-1.83 (m, 1H), 1.94-1.98 (m, 1H), 2.28-2.33 (m, 1H), 2.46-2.48 (m, 2H), 3.92-4.01 (m, 2H), 5.35 (s, 1H), 5.44 (s, 2H), 5.68 (d, *J* = 4.0 Hz, 1H), 6.86 (dd, *J* = 4.0, 2.0 Hz, 1H), 7.04 (td, *J* = 8.0, 2.0 Hz, 1H), 7.29-7.34 (m, 3H), 7.37-7.39 (m, 2H), 7.58 (dd, *J* = 8.0, 2.0 Hz, 1H). ^13^C NMR (150 MHz, CDCl_3_) 170.06, 163.39, 159.82, 158.02, 143.36, 139.39, 136.90, 128.70, 128.64, 128.63, 118.97, 118.81, 116.29, 115.61, 115.44, 109.80, 109.74, 104.82, 92.32, 91.62, 51.63, 46.20, 37.29, 36.20, 35.86, 34.06, 32.36, 31.61, 25.94, 24.67, 21.97, 20.20, 12.08. HRMS-ESI: m/z Calcd for C_33_H_37_FN_2_O_8_Na [M+Na]^+^: 631.2426; Found: 631.2393.

#### 4.2.11 (3*R*,5a*S*,6*R*,8a*S*,9*R*,12*R*,12a*R*)-3,6,9-trimethyldecahydro-12*H*-3,12-epoxy[1,2]dioxepino[4,3-*i*]isochromen-10-yl 3-(5-methoxy-3-(methoxyimino)-2-oxoindolin-1-yl)propanoate (7k)

^1^H NMR (600 MHz, CDCl_3_) δ 0.72-0.95 (m, 7H), 1.18-1.43 (m, 7H), 1.51-1.53 (m, 1H), 1.63-1.70 (m, 2H), 1.80-1.84 (m, 1H), 1.94-1.98 (m, 1H), 2.27-2.33 (m, 1H), 2.46-2.49 (m, 1H), 2.68-2.76 (m, 2H), 3.74 (s, OMe), 3.89-4.01 (m, 2H), 4.22 (s, 3H, NOMe), 5.36 (s, 1H), 5.70 (d, *J* = 4.0 Hz, 1H), 6.78-6.82 (m, 1H), 6.85-6.88 (m, 1H), 7.40-7.42 (m, 1H). ^13^C NMR (150 MHz, CDCl_3_) 170.05, 163.45, 156.92, 156.87, 143.68, 136.98, 117.82, 117.43, 116.43, 116.40, 114.51, 114.50, 109.43, 109.25, 104.60, 92.33, 91.61, 80.08, 64.89, 61.64, 55.99, 46.21, 37.48, 37.37, 37.28, 36.20, 35.73, 35.67, 34.71, 34.06, 32.29, 31.90, 31.62, 26.02, 25.94, 24.67, 21.97, 20.20, 13.17, 12.08. HRMS-ESI: m/z Calcd for C_28_H_36_N_2_O_8_Na [M+Na]^+^: 567.2313; Found: 567.2275.

#### 4.2.12 (3*R*,5a*S*,6*R*,8a*S*,9*R*,12*R*,12a*R*)-3,6,9-trimethyldecahydro-12*H*-3,12-epoxy[1,2]dioxepino[4,3-*i*]isochromen-10-yl 3-(5-methoxy-3-(ethoxyimino)-2-oxoindolin-1-yl)propanoate (7l)

^1^H NMR (600 MHz, CDCl_3_) δ 0.72-0.96 (m, 7H), 1.20-1.42 (m, 10H), 1.52-1.56 (m, 1H), 1.63-1.70 (m, 2H), 1.80-1.84 (m, 1H), 1.94-1.98 (m, 1H), 2.23-2.33 (m, 1H), 2.46-2.48 (m, 1H), 2.68-2.76 (m, 2H), 3.74 (s, 3H, OMe), 3.89-4.03 (m, 2H), 4.48 (q, *J* = 8.0 Hz, 2H), 5.36 (s, 1H), 5.70 (d, *J* = 4.0 Hz, 1H), 6.78-6.80 (m, 1H), 6.86-6.88 (m, 1H), 7.51-7.54 (m, 1H). ^13^C NMR (150 MHz, CDCl_3_) 170.08, 163.57, 156.83, 143.63, 136.87, 117.04, 116.86, 114.88, 109.33, 104.60, 92.31, 91.62, 80.08, 73.10, 55.94, 51.54, 45.21, 37.28, 36.21, 35.64, 34.06, 32.30, 31.63, 26.96, 24.67, 21.98, 20.21, 14.72, 12.09. HRMS-ESI: m/z Calcd for C_29_H_38_N_2_O_8_Na [M+Na]^+^: 581.2470; Found: 581.2442.

#### 4.2.13 (3*R*,5a*S*,6*R*,8a*S*,9*R*,12*R*,12a*R*)-3,6,9-trimethyldecahydro-12*H*-3,12-epoxy[1,2]dioxepino[4,3-*i*]isochromen-10-yl 4-(3-(methoxyimino)-2-oxoindolin-1-yl)butanoate (7m)

^1^H NMR (600 MHz, CDCl_3_) δ 0.77-0.96 (m, 7H), 1.19-1.43 (m, 7H), 1.54-1.58 (m, 1H), 1.64-1.73 (m, 2H), 1.81-1.84 (m, 1H), 1.91-1.99 (m, 3H), 2.28-2.33 (m, 1H), 2.49-2.61 (m, 3H), 3.67-3.79 (m, 2H), 4.22 (s, 3H, NOMe), 5.39 (s, 1H), 5.73 (d, *J* = 4.0 Hz, 1H), 6.92 (d, *J* = 4.0 Hz, 1H), 6.98 (t, *J* = 4.0 Hz, 1H), 7.34 (t, *J* = 4.0 Hz, 1H), 7.88 (d, *J* = 4.0 Hz, 1H). ^13^C NMR (150 MHz, CDCl_3_) 171.71, 163.80, 143.68, 143.61, 132.77, 127.95, 122.93, 116.76, 108.97, 104.49, 92.13, 91.60, 80.10, 64.75, 61.86, 45.23, 38.94, 37.29, 36.22, 34.09, 31.71, 31.03, 26.96, 24.89, 22.36, 22.00, 20.22, 12.16. HRMS-ESI: m/z Calcd for C_28_H_36_N_2_O_8_Na [M+Na]^+^: 551.2364; Found: 551.2318.

#### 4.2.14 (3*R*,5a*S*,6*R*,8a*S*,9*R*,12*R*,12a*R*)-3,6,9-trimethyldecahydro-12*H*-3,12-epoxy[1,2]dioxepino[4,3-*i*]isochromen-10-yl 4-(3-(ethoxyimino)-2-oxoindolin-1-yl)butanoate (7n)

^1^H NMR (600 MHz, CDCl_3_) δ 0.77-0.96 (m, 7H), 1.19-1.27 (m, 2H), 1.31-1.42 (m, 8H), 1.52-1.57 (m, 1H), 1.64-1.72 (m, 2H), 1.81-1.83 (m, 1H), 1.91-1.99 (m, 3H), 2.28-2.33 (m, 1H), 2.40-2.52 (m, 3H), 3.70-3.80 (m, 2H), 4.48 (q, *J* = 4.0 Hz, 1H), 5.38 (s, 1H), 5.74 (d, *J* = 4.0 Hz, 1H), 6.92 (d, *J* = 4.0 Hz, 1H), 6.98 (t, *J* = 4.0 Hz, 1H), 7.34 (t, *J* = 8.0 Hz, 1H), 7.90 (d, *J* = 8.0 Hz, 1H). ^13^C NMR (150 MHz, CDCl_3_) 171.73, 163.64, 143.86, 143.64, 132.68, 127.85, 122.89, 116.69, 108.90, 104.49, 92.13, 91.60, 80.10, 72.99, 61.60, 45.23, 38.91, 37.29, 36.22, 34.09, 31.71, 31.06, 26.94, 24.89, 22.38, 22.00, 20.22, 14.73, 12.15. HRMS-ESI: m/z Calcd for C_29_H_38_N_2_O_8_Na [M+Na]^+^: 565.2520; Found: 565.2484.

#### 4.2.15 (3*R*,5a*S*,6*R*,8a*S*,9*R*,12*R*,12a*R*)-3,6,9-trimethyldecahydro-12*H*-3,12-epoxy[1,2]dioxepino[4,3-*i*]isochromen-10-yl 4-(3-[(benzyloxy)imino)-2-oxoindolin-1-yl]butanoate (7o)

^1^H NMR (600 MHz, CDCl_3_) δ 0.77-0.96 (m, 7H), 1.18-1.42 (m, 7H), 1.53-1.57 (m, 1H), 1.64-1.72 (m, 2H), 1.80-1.83 (m, 1H), 1.90-1.98 (m, 3H), 2.27-2.33 (m, 1H), 2.39-2.52 (m, 3H), 3.65-3.78 (m, 2H), 5.37 (s, 1H), 5.46 (s, 2H), 5.72 (d, *J* = 8.0 Hz, 1H), 6.91-6.95 (m, 2H), 7.28-7.33 (m, 4H), 7.37-7.39 (m, 2H), 7.86 (d, *J* = 4.0 Hz, 1H). ^13^C NMR (150 MHz, CDCl_3_) 171.72, 163.63, 143.98, 143.69, 136.27, 132.82, 128.52, 128.46, 128.44, 128.35, 128.19, 128.12, 122.99, 116.81, 108.96, 104.50, 92.16, 91.61, 80.11, 79.36, 61.67, 45.23, 38.96, 37.29, 36.22, 34.09, 31.71, 31.04, 25.95, 24.89, 22.36, 22.01, 20.22, 12.16. HRMS-ESI: m/z Calcd for C_34_H_40_N_2_O_8_Na [M+Na]^+^: 627.2647; Found: 627.2617.

#### 4.2.16 (3*R*,5a*S*,6*R*,8a*S*,9*R*,12*R*,12a*R*)-3,6,9-trimethyldecahydro-12*H*-3,12-epoxy[1,2]dioxepino[4,3-*i*]isochromen-10-yl 4-(3-(methoxyimino)-5-fluoro-2-oxoindolin-1-yl)butanoate (7p)

^1^H NMR (600 MHz, CDCl_3_) δ 0.77-0.96 (m, 7H), 1.20-1.42 (m, 7H), 1.53-1.57 (m, 1H), 1.64-1.72 (m, 2H), 1.80-1.84 (m, 1H), 1.91-1.98 (m, 3H), 2.26-2.33 (m, 1H), 2.37-2.50 (m, 3H), 3.66-3.77 (m, 2H), 4.22 (s, 3H, NOMe), 5.37 (s, 1H), 5.74 (d, *J* = 8.0 Hz, 1H), 6.80 (d, *J* = 4.0 Hz, 1H), 7.14 (d, *J* = 4.0 Hz, 1H), 7.71 (s, 1H). ^13^C NMR (150 MHz, CDCl_3_) 171.73, 163.66, 143.80, 141.43, 133.03, 132.49, 128.09, 116.78, 108.71, 104.49, 92.11, 91.80, 80.10, 64.89, 51.57, 46.23, 38.97, 37.29, 36.22, 34.09, 31.71, 31.05, 25.94, 24.68, 22.38, 22.00, 20.96, 20.22, 12.14. HRMS-ESI: m/z Calcd for C_28_H_38_FN_2_O_9_ [M+H_3_O]^+^: 565.2455; Found: 565.2496.

#### 4.2.17 (3*R*,5a*S*,6*R*,8a*S*,9*R*,12*R*,12a*R*)-3,6,9-trimethyldecahydro-12*H*-3,12-epoxy[1,2]dioxepino[4,3-*i*]isochromen-10-yl 4-(3-(ethoxyimino)-5-fluoro-2-oxoindolin-1-yl)butanoate (7q)

^1^H NMR (600 MHz, CDCl_3_) δ 0.77-0.96 (m, 7H), 1.19-1.44 (m, 10H), 1.56-1.58 (m, 1H), 1.64-1.73 (m, 2H), 1.81-1.84 (m, 1H), 1.90-1.97 (m, 3H), 2.28-2.33 (m, 1H), 2.39-2.51 (m, 3H), 3.65-3.79 (m, 2H), 4.60 (q, *J* = 4.0 Hz, 2H), 5.38 (s, 1H), 5.74 (d, *J* = 4.0 Hz, 1H), 6.80-6.91 (m, 1H), 7.03-7.09 (m, 1H), 7.62-7.66 (m, 1H). ^13^C NMR (150 MHz, CDCl_3_) 176.29, 171.72, 163.42, 159.83, 158.04, 143.09, 139.60, 118.91, 118.76, 118.69, 118.53, 116.36, 116.24, 116.03, 115.42, 115.25, 109.64, 109.50, 109.30, 104.32, 92.18, 91.82, 80.10, 73.44, 73.36, 51.86, 45.22, 39.07, 39.04, 37.49, 37.30, 36.35, 36.21, 34.21, 34.08, 31.71, 30.92, 30.62, 25.93, 24.89, 22.83, 22.26, 22.00, 20.22, 14.71, 13.18, 12.14. HRMS-ESI: m/z Calcd for C_29_H_37_FN_2_O_8_Na [M+Na]^+^: 583.2426; Found: 583.2405.

#### 4.2.18 (3*R*,5a*S*,6*R*,8a*S*,9*R*,12*R*,12a*R*)-3,6,9-trimethyldecahydro-12*H*-3,12-epoxy[1,2]dioxepino[4,3-*i*]isochromen-10-yl 4-(3-[(benzyloxy)imino)-5-fluoro-2-oxoindolin-1-yl]butanoate (7r)

^1^H NMR (600 MHz, CDCl_3_) δ 0.77-0.96 (m, 7H), 1.21-1.42 (m, 7H), 1.54-1.58 (m, 1H), 1.64-1.72 (m, 2H), 1.81-1.85 (m, 1H), 1.90-1.98 (m, 3H), 2.28-2.33 (m, 1H), 2.39-2.52 (m, 3H), 3.64-3.80 (m, 2H), 5.38 (s, 1H), 5.46 (s, 2H), 5.74 (d, *J* = 4.0 Hz, 1H), 6.88 (dd, *J* = 8.0, 4.0 Hz, 1H), 7.06 (td, *J* = 8.0, 2.0 Hz, 1H), 7.58 (dd, *J* = 8.0, 2.0 Hz, 1H). ^13^C NMR (150 MHz, CDCl_3_) 171.84, 163.27, 159.61, 158.02, 143.68, 139.73, 136.95, 128.69, 128.62, 128.52, 119.18, 119.02, 116.27, 116.21, 115.62, 115.45, 109.71, 109.66, 104.62, 92.18, 91.82, 80.10, 79.89, 51.66, 45.22, 39.07, 37.30, 36.21, 34.09, 31.71, 30.92, 26.94, 24.69, 22.25, 22.01, 20.22, 12.16. HRMS-ESI: m/z Calcd for C_34_H_39_FN_2_O_8_Na [M+Na]^+^: 645.2583; Found: 645.2561.

#### 4.2.19 (3*R*,5a*S*,6*R*,8a*S*,9*R*,12*R*,12a*R*)-3,6,9-trimethyldecahydro-12*H*-3,12-epoxy[1,2]dioxepino[4,3-*i*]isochromen-10-yl 4-(5-methoxy-3-(methoxyimino)-2-oxoindolin-1-yl)butanoate (7s)

^1^H NMR (600 MHz, CDCl_3_) δ 0.77-0.96 (m, 7H), 1.19-1.42 (m, 7H), 1.54-1.57 (m, 1H), 1.64-1.73 (m, 2H), 1.81-1.85 (m, 1H), 1.90-1.99 (m, 3H), 2.28-2.33 (m, 1H), 2.37-2.52 (m, 3H), 3.63-3.77 (m, 5H), 4.42 (s, 3H, NOMe), 5.38 (s, 1H), 5.74 (d, *J* = 8.0 Hz, 1H), 6.79-6.91 (m, 2H), 7.50 (d, *J* = 2.0 Hz, 1H). ^13^C NMR (150 MHz, CDCl_3_) 171.72, 163.36, 156.82, 143.89, 137.41, 117.77, 116.31, 114.45, 109.47, 104.49, 92.13, 91.80, 80.09, 64.80, 56.00, 51.57, 45.23, 38.99, 37.29, 36.22, 34.09, 31.71, 31.03, 25.95, 24.69, 22.35, 22.00, 20.22, 12.15. HRMS-ESI: m/z Calcd for C_29_H_38_N_2_O_9_Na [M+Na]^+^: 581.2470; Found: 581.2440.

#### 4.2.20 (3*R*,5a*S*,6*R*,8a*S*,9*R*,12*R*,12a*R*)-3,6,9-trimethyldecahydro-12*H*-3,12-epoxy[1,2]dioxepino[4,3-*i*]isochromen-10-yl 4-(3-(ethoxyimino)-5-methoxy-2-oxoindolin-1-yl)butanoate (7t)

^1^H NMR (600 MHz, CDCl_3_) δ 0.77-0.96 (m, 7H), 1.21-1.43 (m, 10H), 1.52-1.57 (m, 1H), 1.64-1.73 (m, 2H), 1.81-1.84 (m, 1H), 1.91-1.98 (m, 3H), 2.28-2.33 (m, 1H), 2.38-2.52 (m, 3H), 3.64-3.78 (m, 5H), 4.48 (q, *J* = 8.0 Hz, 2H), 5.38 (s, 1H), 5.74 (d, *J* = 8.0 Hz, 1H), 6.76-6.90 (m, 2H), 7.53 (d, *J* = 2.0 Hz, 1H). ^13^C NMR (150 MHz, CDCl_3_) 171.75, 163.30, 156.78, 143.72, 137.29, 117.29 116.47, 114.65, 109.38, 104.30, 92.12, 91.61, 80.10, 73.04, 55.95, 51.57, 45.23, 38.97, 37.29, 36.22, 34.09, 31.72, 31.05, 25.95, 22.35, 22.01, 20.22, 14.74, 12.15. HRMS-ESI: m/z Calcd for C_30_H_40_N_2_O_9_Na [M+Na]^+^: 595.2626; Found: 595.2605.

#### 4.2.21 (3*R*,5a*S*,6*R*,8a*S*,9*R*,12*R*,12a*R*)-3,6,9-trimethyldecahydro-12*H*-3,12-epoxy[1,2]dioxepino[4,3-*i*]isochromen-10-yl 4-(3-[(benzyloxy)imino)-5-methoxy-2-oxoindolin-1-yl]butanoate (7u)

^1^H NMR (600 MHz, CDCl_3_) δ 0.77-0.96 (m, 7H), 1.18-1.42 (m, 7H), 1.53-1.57 (m, 1H), 1.63-1.72 (m, 2H), 1.80-1.84 (m, 1H), 1.90-1.97 (m, 3H), 2.27-2.33 (m, 1H), 2.38-2.50 (m, 3H), 3.63-3.77 (m, 5H), 5.37 (s, 1H), 5.54 (s, 2H), 5.72 (d, *J* = 4.0 Hz, 1H), 6.75-6.89 (m, 2H), 7.27-7.38 (m, 5H), 7.49 (d, *J* = 2.0 Hz, 1H). ^13^C NMR (150 MHz, CDCl_3_) 171.74, 163.38, 156.79, 144.33, 137.41, 136.29, 128.60, 128.49, 128.44, 128.35, 128.33, 117.66, 116.38, 114.71, 109.48, 104.30, 92.14, 91.61, 80.10, 79.31, 55.87, 51.57, 45.23, 39.02, 37.29, 36.22, 34.09, 31.72, 31.04, 25.95, 24.89, 22.35, 22.01, 20.22, 12.15. HRMS-ESI: m/z Calcd for C_35_H_42_N_2_O_9_Na [M+Na]^+^: 657.2783; Found: 657.2741.

### 4.3 *In vitro* antiproliferative activity evaluation

K562 CM-0130 (purchased from Procell), K562/ADR AW-CELLS-H0187 (purchased from AnWei-sci) and CCRF-CEM CL-0058 (purchased from Procell) leukemia cell lines (2×10^3^) were plated in each well of a 96-well plate and were allowed to adhere and spread for 24 h at 37°C in 5% CO_2_ for attachment. Medium in wells was replaced after 24 h with fresh medium containing the tested compounds in DMSO (1% *v/v*) and incubated for 72 h. The ART-isatin hybrids 7a-u along with the positive control Adriamycin and Vorinostat were added to a final concentration of 100 *µ*M, and the cells were cultured for 24 h at 37°C. 3-(4,5-dimethyl-2-thiazolyl)-2,5-diphenyltetrazolium bromide (MTT) solution (10 *µ*L) was added to each well, and the cultures were incubated for an additional 4 h at 37°C. A further 100 *µ*L of MTT solution was added and incubation continued overnight at 37°C. The absorbance at 540 nm was determined in each well with a 96-well plate reader. The growth of the treated cells was compared with that of untreated cells. Half maximum inhibitory concentration (IC_50_) is a measurement of the efficacy of a substance in inhibiting a specific biological or biochemical process. For a drug, IC_50_ represents the drug concentration required to inhibit the activity of tumor cells by 50% *in vitro*, and it is the most important parameter to compare the efficacy of a drug with that of similar drugs. In this experiment, the dose-response curve constructed by Spss22.0 software was used to calculate the inhibitory effect of the hybrid (0 *μ*M-100 *μ*M) on the activity of leukemia cell lines. Each experiment was performed independently in triplicate with three tests in each, and results were reported as mean inhibitory concentration IC_50_.

### 4.4 Pharmacokinetic profiles determination

CD-1 mice mice (20-25 g) were used in the pharmacokinetic study, and each treatment group had 3 mice which were dosed with hybrid 7d suspension at 30 mg/kg by single intravenous (iv) administration. Compounds were suspended in 0.5% CMC for iv, and blood was collected from the jugular vein of each mouse at the following time points: 0.25, 0.5, 1, 2, 4, 6, 8 and 24 h after iv administration. Total area under the concentration time curve (AUC), the elimination half-time (t_1/2_), the peak concentration (C_max_) and the time to reach peak concentration (T_max_) of samples were determined directly from the experimental data using WinNonlin V6.2.1.

## Data availability statement

The original contributions presented in the study are included in the article/**Supplementary Material**. Further inquiries can be directed to the corresponding author.

## Author contributions

All authors listed have made a substantial, direct, and intellectual contribution to the work and approved it for publication.
