# Supplementary material for: Development and evaluation of novel artemisinin-isatin hybrids with potential anti-leukemic cytotoxicity
Source: Front Oncol. 2023 Apr 14;13:1112369. doi: 10.3389/fonc.2023.1112369 (PMC10140581; doi:10.3389/fonc.2023.1112369)
Supplement: Supplementary file 1 [file DataSheet_1.docx]

**Development and evaluation of novel artemisinin-isatin hybrids with potential anti-leukemic cytotoxicity**

Peng Wang^1#^, Zhe Zhang^2#^, Wei Cao^3^, Xuan Zhang^4*^

1 Department of Critical Care Unit, Shandong Provincial Hospital Affiliated to Shandong First Medical University, Shandong, China

2 Department of Tumor Radiotherapy, Shandong Provincial Hospital Affiliated to Shandong First Medical University, Shandong, China

3 Department of Nephrology, The First Affiliated Hospital of Shandong First Medical University & Provincial Qianfoshan Hospital, Shandong Institute of Nephrology, Shandong, China

4 Department of Geriatric Respiratory Disease, Shandong Provincial Hospital Affiliated to Shandong First Medical University, Shandong, China

# These authors contributed equally to this work.

Corresponding author: [csdzx@126.com(X](mailto:csdzx@126.com(X). Zhang)

(3*R*,5a*S*,6*R*,8a*S*,9*R*,12*R*,12a*R*)-3,6,9-trimethyldecahydro-12*H*-3,12-epoxy[1,2]dioxepino[4,3-*i*]isochromen-10-yl 2-(3-(methoxyimino)-2-oxoindolin-1-yl)acetate (**7a**)

^1^H NMR (600 MHz, CDCl_3_) δ 0.86-1.03 (m, 7H), 1.27-1.39 (m, 3H), 1.43-1.54 (m, 4H), 1.62-1.66 (m, 1H), 1.71-1.74 (m, 1H), 1.77-1.80 (m, 1H), 1.89-1.923 (m, 1H), 2.03-2.07 (m, 1H), 2.37-2.42 (m, 1H), 2.57-2.61 (m, 1H), 4.32 (s, 3H, NOMe), 4.50 (d, *J* = 12.0 Hz, 1H), 4.72 (d, *J* = 12.0 Hz, 1H), 5.44 (s, 1H), 5.82 (d, *J* = 4.0 Hz, 1H), 6.76 (d, *J* = 4.0 Hz, 1H), 7.10 (t, *J* = 4.0 Hz, 1H), 7.38 (t, *J* = 4.0 Hz, 1H), 8.00 (d, *J* = 4.0 Hz, 1H). ^13^C NMR (150 MHz, CDCl_3_) 164.41, 161.66, 141.29, 141.11, 130.82, 126.08, 121.48, 113.93, 106.93, 102.66, 91.41, 89.63, 78.15, 62.97, 49.38, 43.28, 39.24, 36.34, 34.27, 32.12, 29.89, 23.97, 22.66, 20.04, 18.27, 10.19. HRMS-ESI: m/z Calcd for C_26_H_32_N_2_O_8_Na [M+Na]^+^: 523.2051; Found: 523.2006.


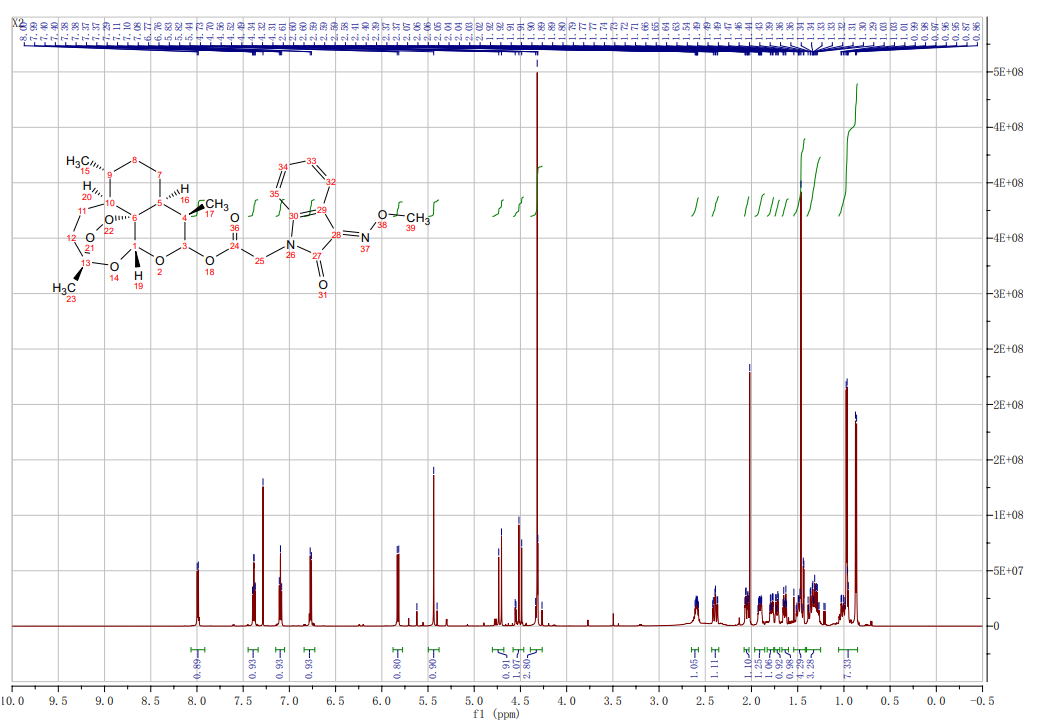


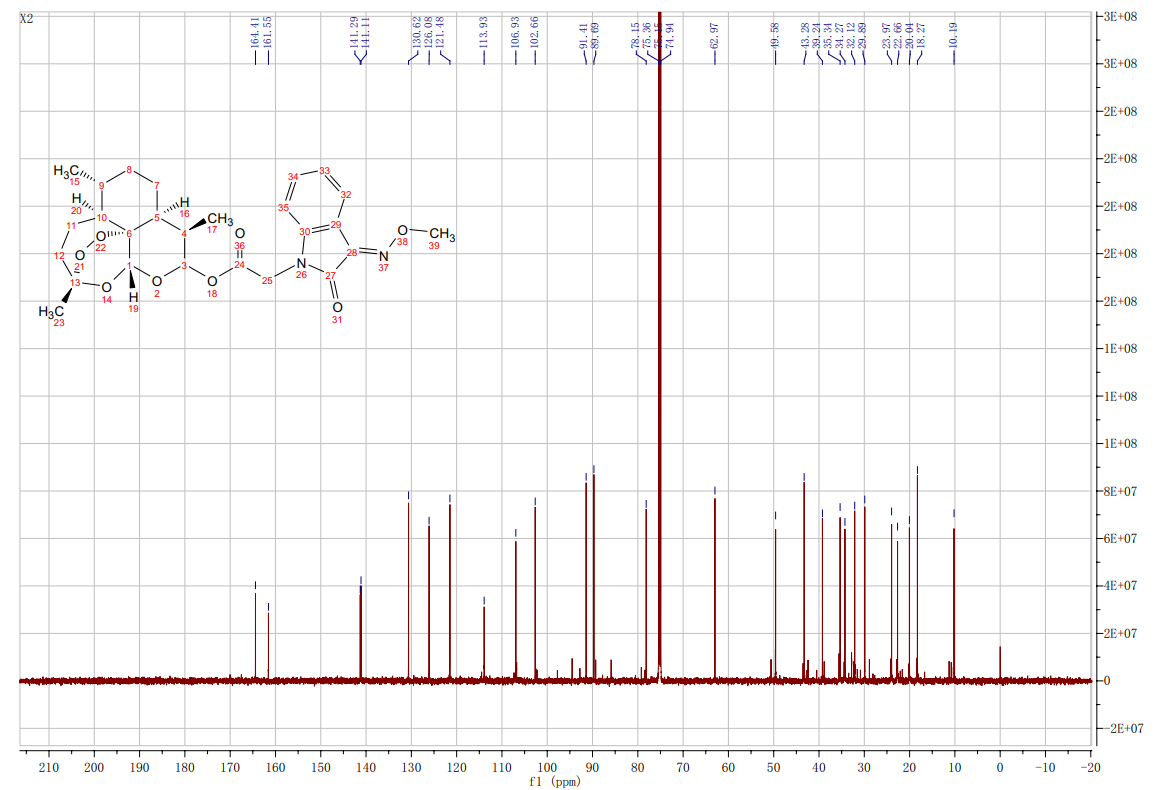


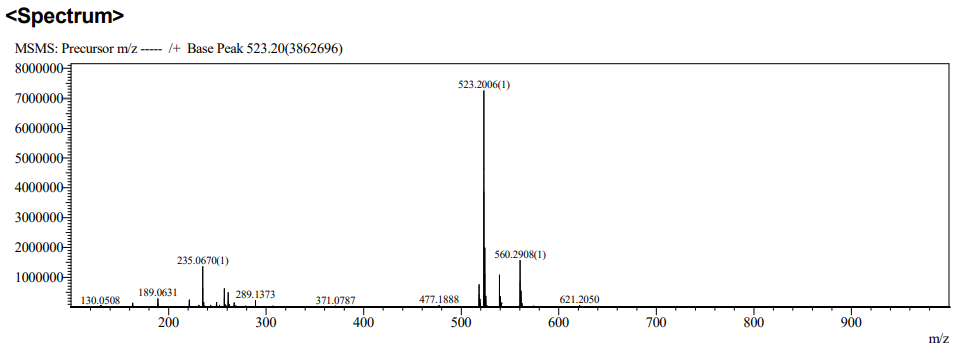


(3*R*,5a*S*,6*R*,8a*S*,9*R*,12*R*,12a*R*)-3,6,9-trimethyldecahydro-12*H*-3,12-epoxy[1,2]dioxepino[4,3-*i*]isochromen-10-yl 2-(3-((benzyloxy)imino)-2-oxoindolin-1-yl)acetate (**7b**)

^1^H NMR (600 MHz, CDCl_3_) δ 0.87-1.03 (m, 7H), 1.29-1.39 (m, 3H), 1.44-1.53 (m, 4H), 1.64-1.68 (m, 1H), 1.72-1.80 (m, 2H), 1.90-1.92 (m, 1H), 2.04-2.08 (m, 1H), 2.37-2.43 (m, 1H), 2.60-2.62 (m, 1H), 4.50 (d, *J* = 12.0 Hz, 2H), 4.72 (d, *J* = 12.0 Hz, 2H), 5.45 (s, 1H), 5.68 (s, 2H), 5.84 (d, *J* = 4.0 Hz, 1H), 6.76 (d, *J* = 4.0 Hz, 1H), 7.06 (t, *J* = 4.0 Hz, 1H), 7.36-7.43 (m, 4H), 7.46-7.48 (m, 2H), 7.98 (d, *J* = 4.0 Hz, 1H). ^13^C NMR (150 MHz, CDCl_3_) 166.33, 163.80, 143.64, 143.02, 136.14, 132.67, 128.64, 128.51, 128.48, 128.16, 123.46, 116.89, 108.82, 104.38, 93.33, 91.61, 80.06, 79.82, 51.49, 45.19, 41.16, 37.25, 36.18, 34.03, 31.81, 26.88, 24.67, 21.96, 20.18, 12.10. HRMS-ESI: m/z Calcd for C_28_H_36_N_2_O_8_Na [M+Na]^+^: 599.2364; Found: 599.2326.


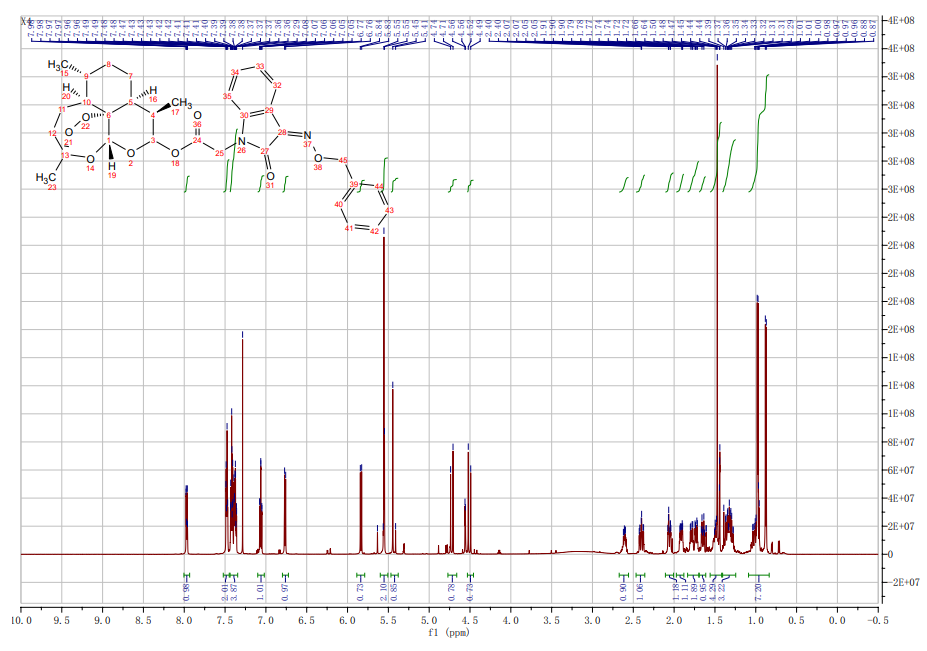


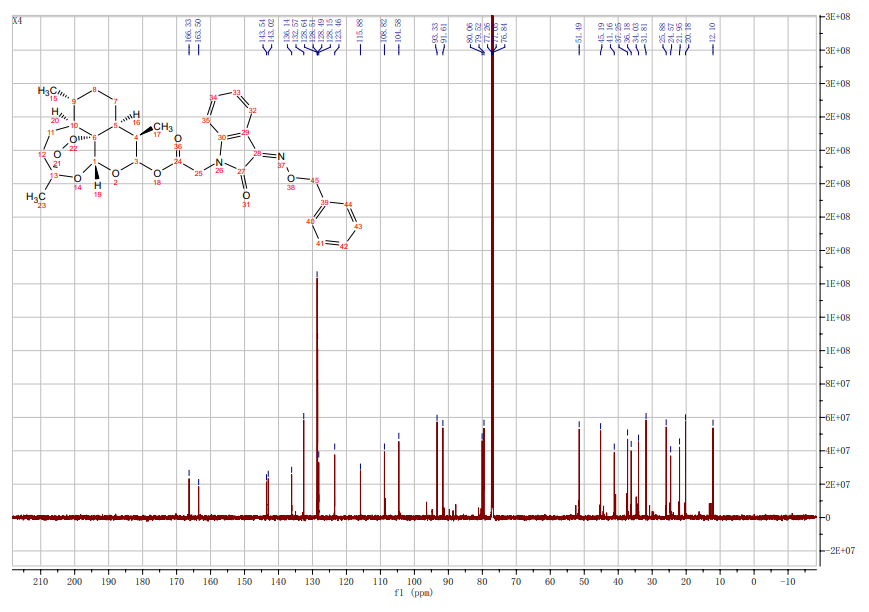


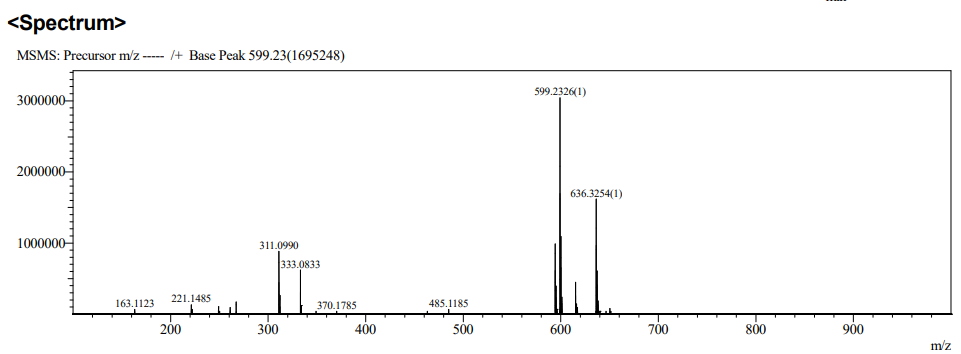


(3*R*,5a*S*,6*R*,8a*S*,9*R*,12*R*,12a*R*)-3,6,9-trimethyldecahydro-12*H*-3,12-epoxy[1,2]dioxepino[4,3-*i*]isochromen-10-yl 2-(5-fluoro-3-(methoxyimino)-2-oxoindolin-1-yl)acetate (**7c**)

^1^H NMR (600 MHz, CDCl_3_) δ 0.72-0.92 (m, 7H), 1.09-1.42 (m, 7H), 1.47-1.64 (m, 3H), 1.74-1.78 (m, 1H), 1.89-1.92 (m, 1H), 2.14-2.19 (m, 1H), 2.31-2.34 (m, 1H), 4.16 (s, 3H, NOMe), 4.56-4.64 (m, 2H), 5.41 (s, 1H), 5.66 (d, *J* = 8.0 Hz, 1H), 6.96-6.99 (m, 1H), 7.12-7.16 (m, 1H), 7.62-7.64 (m, 1H). HRMS-ESI: m/z Calcd for C_26_H_31_FN_2_O_8_Na [M+Na]^+^: 541.1957; Found: 541.1916.


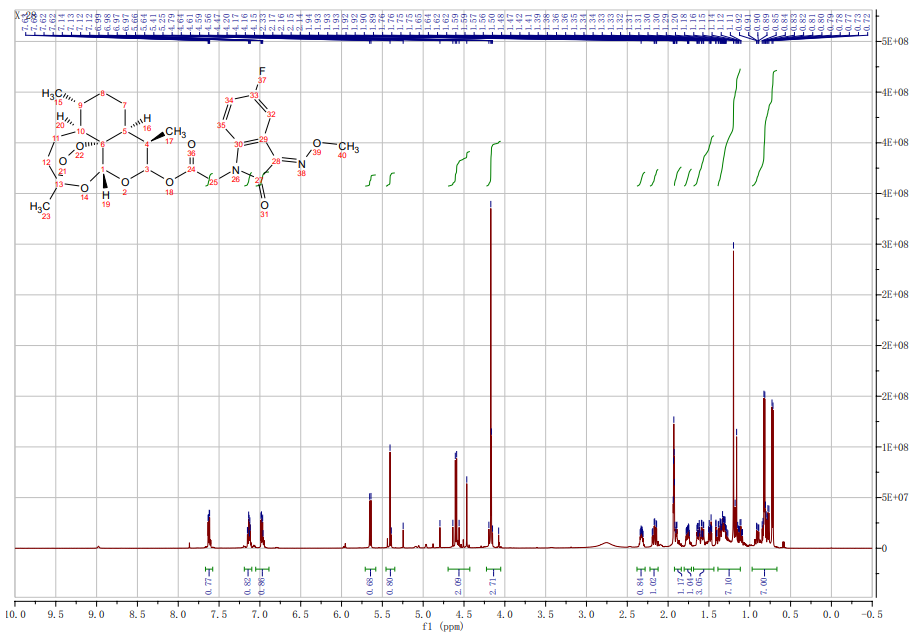


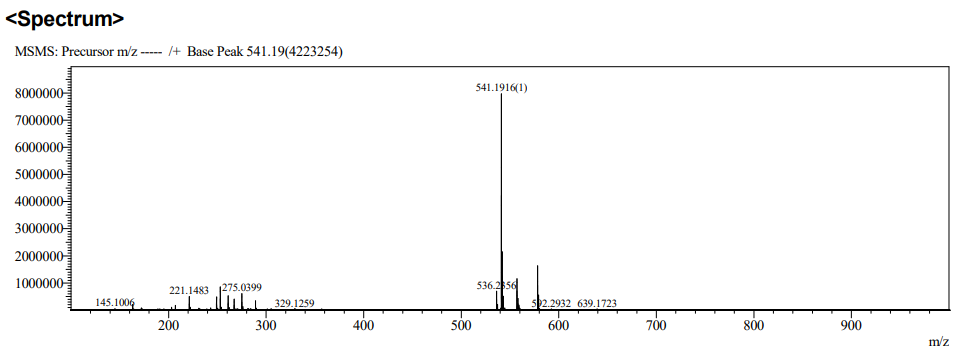


(3*R*,5a*S*,6*R*,8a*S*,9*R*,12*R*,12a*R*)-3,6,9-trimethyldecahydro-12*H*-3,12-epoxy[1,2]dioxepino[4,3-*i*]isochromen-10-yl 2-(5-fluoro-3-(methoxyimino)-2-oxoindolin-1-yl)acetate (**7d**)

^1^H NMR (600 MHz, CDCl_3_) δ 0.78-0.96 (m, 7H), 1.19-1.41 (m, 7H), 1.54-1.57 (m, 1H), 1.63-1.71 (m, 2H), 1.81-1.83 (m, 1H), 1.94-1.98 (m, 1H), 2.28-2.34 (m, 1H), 2.49-2.53 (m, 1H), 3.74 (s, 3H, OMe), 4.25 (s, 3H, NOMe), 4.36 (d, *J* = 12.0 Hz, 1H), 4.62 (d, *J* = 12.0 Hz, 1H), 5.36 (s, 1H), 5.74 (d, *J* = 8.0 Hz, 1H), 6.60 (d, *J* = 4.0 Hz, 1H), 6.84 (dd, *J* = 4.0, 2.0 Hz, 1H), 7.52 (d, *J* = 2.0 Hz, 1H). ^13^C NMR (150 MHz, CDCl_3_) 166.39, 163.41, 156.10, 143.48, 136.79, 117.69, 116.38, 114.42, 109.33, 104.86, 93.27, 91.60, 80.06, 64.94, 56.00, 51.40, 45.19, 41.25, 37.25, 36.18, 34.03, 31.80, 26.89, 24.67, 21.96, 20.18, 12.11. HRMS-ESI: m/z Calcd for C_26_H_31_FN_2_O_8_Na [M+Na]^+^: 541.1957; Found: 541.1916.


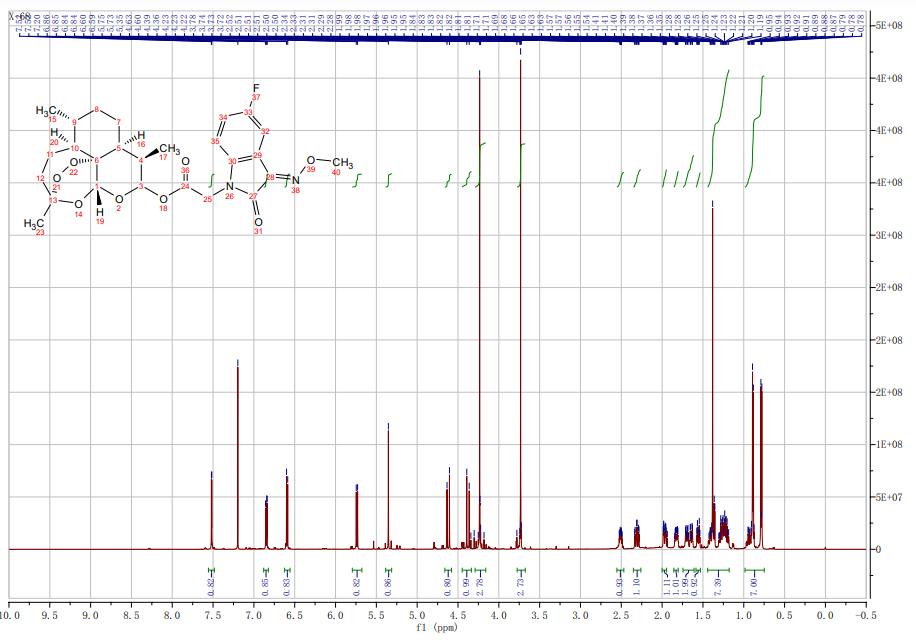


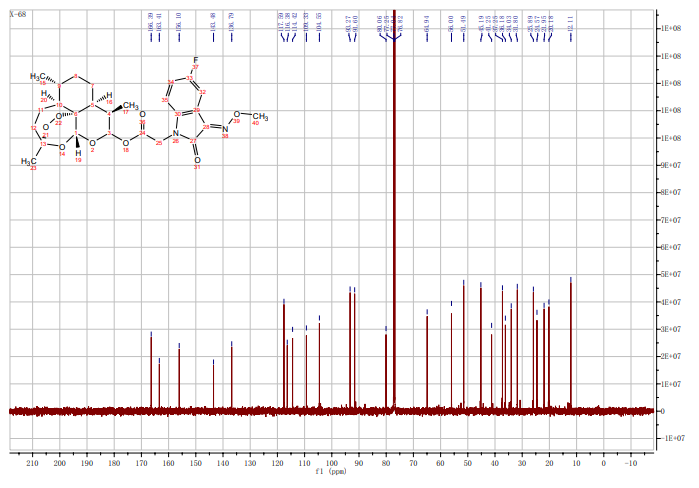


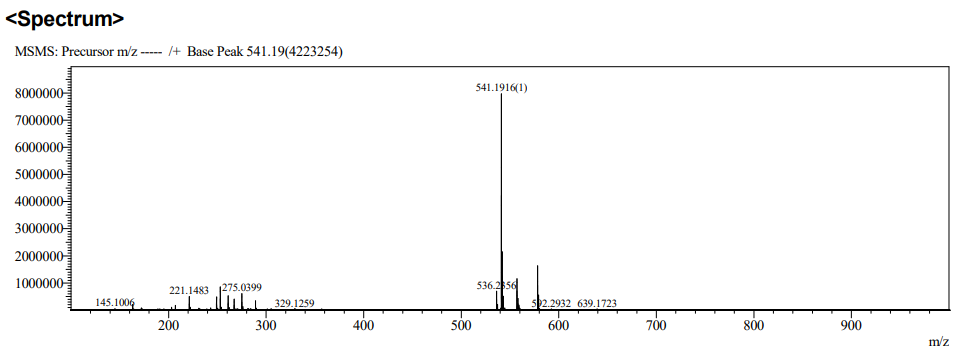


(3*R*,5a*S*,6*R*,8a*S*,9*R*,12*R*,12a*R*)-3,6,9-trimethyldecahydro-12*H*-3,12-epoxy[1,2]dioxepino[4,3-*i*]isochromen-10-yl 3-(3-(methoxyimino)-2-oxoindolin-1-yl)propanoate (**7e**)

^1^H NMR (600 MHz, CDCl_3_) δ 0.70-0.96 (m, 7H), 1.19-1.30 (m, 3H), 1.36-1.45 (m, 4H), 1.52-1.55 (m, 1H), 1.63-1.69 (m, 2H), 1.80-1.83 (m, 1H), 1.94-1.98 (m, 1H), 2.27-2.33 (m, 1H), 2.46-2.48 (m, 1H), 2.76 (t, *J* = 4.0 Hz, 1H), 3.92-4.06 (m, 2H), 4.22 (s, 3H, NOMe), 5.36 (s, 1H), 5.70 (d, *J* = 4.0 Hz, 1H), 6.90 (d, *J* = 4.0 Hz, 1H), 6.98 (t, *J* = 4.0 Hz, 1H), 7.34 (t, *J* = 4.0 Hz, 1H), 7.88 (d, *J* = 4.0 Hz, 1H). ^13^C NMR (150 MHz, CDCl_3_) 169.99, 163.66, 143.41, 143.27, 132.89, 127.99, 123.05, 116.84, 108.94, 104.80, 92.34, 91.61, 80.07, 64.80, 61.83, 46.20, 37.28, 36.20, 35.68, 34.05, 32.25, 31.82, 25.94, 24.67, 21.97, 20.20, 12.08. HRMS-ESI: m/z Calcd for C_27_H_34_N_2_O_8_Na [M+Na]^+^: 537.2207; Found: 537.2170.


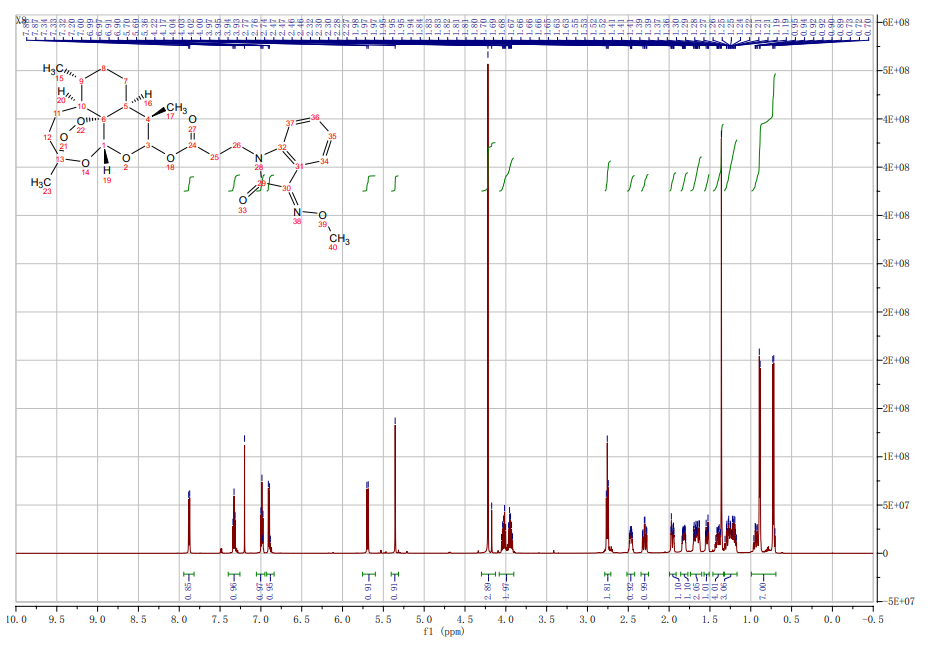


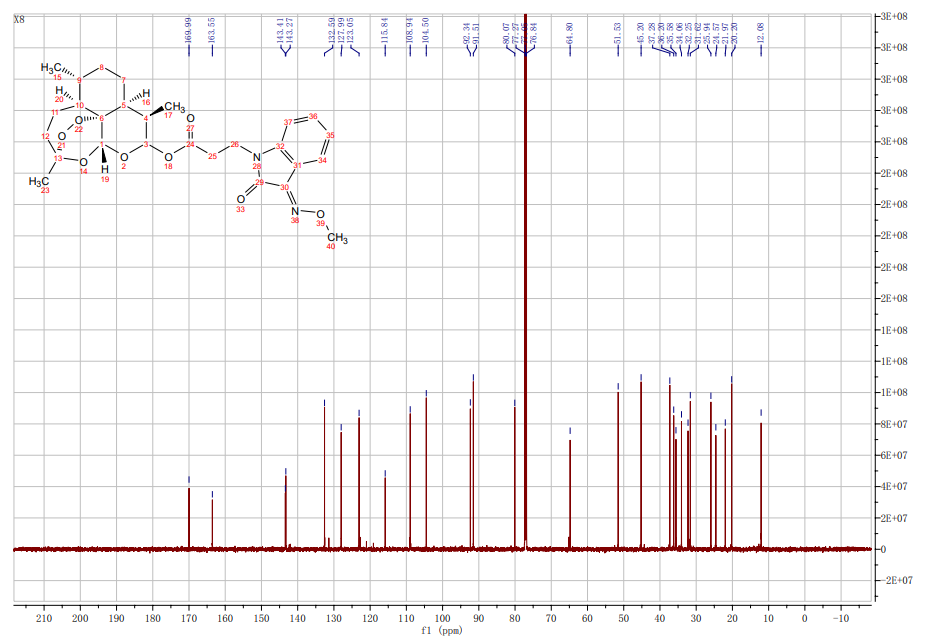


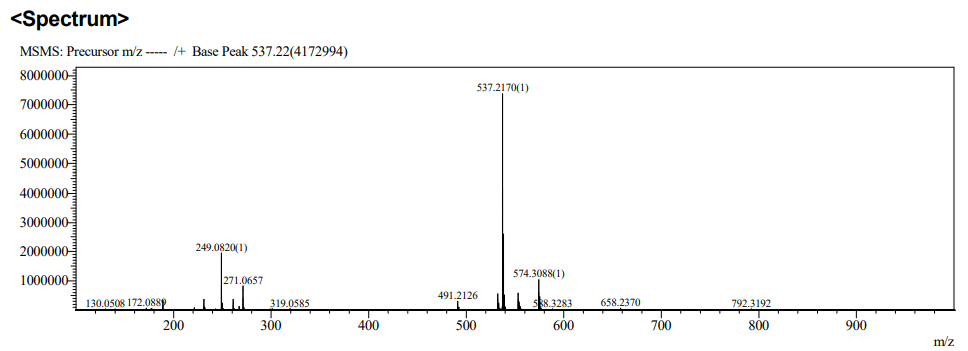


(3*R*,5a*S*,6*R*,8a*S*,9*R*,12*R*,12a*R*)-3,6,9-trimethyldecahydro-12*H*-3,12-epoxy[1,2]dioxepino[4,3-*i*]isochromen-10-yl 3-(3-(ethoxyimino)-2-oxoindolin-1-yl)propanoate (**7f**)

^1^H NMR (600 MHz, CDCl_3_) δ 0.72-0.96 (m, 7H), 1.20-1.28 (m, 3H), 1.36-1.41 (m, 7H), 1.51-1.69 (m, 3H), 1.80-1.83 (m, 1H), 1.94-1.98 (m, 1H), 2.27-2.33 (m, 1H), 2.46-2.48 (m, 1H), 2.76 (t, *J* = 4.0 Hz, 2H), 3.93-4.04 (m, 2H), 4.48 (q, *J* = 4.0 Hz, 2H), 5.36 (s, 1H), 5.70 (d, *J* = 4.0 Hz, 1H), 6.88 (d, *J* = 8.0 Hz, 1H), 6.98-7.01 (m, 1H), 7.32 (d, *J* = 8.0 Hz, 1H), 7.89-7.91 (m, 1H). ^13^C NMR (150 MHz, CDCl_3_) 170.03, 163.71, 143.14, 132.41, 132.36, 127.90, 123.08, 123.01, 116.98, 108.87, 104.61, 92.33, 91.62, 80.08, 73.11, 73.06, 61.61, 45.21, 37.27, 36.20, 36.05, 34.70, 34.06, 32.28, 31.89, 31.62, 26.94, 24.67, 21.97, 20.20, 14.72, 12.08. HRMS-ESI: m/z Calcd for C_28_H_36_N_2_O_8_Na [M+Na]^+^: 551.2364; Found: 551.2344.


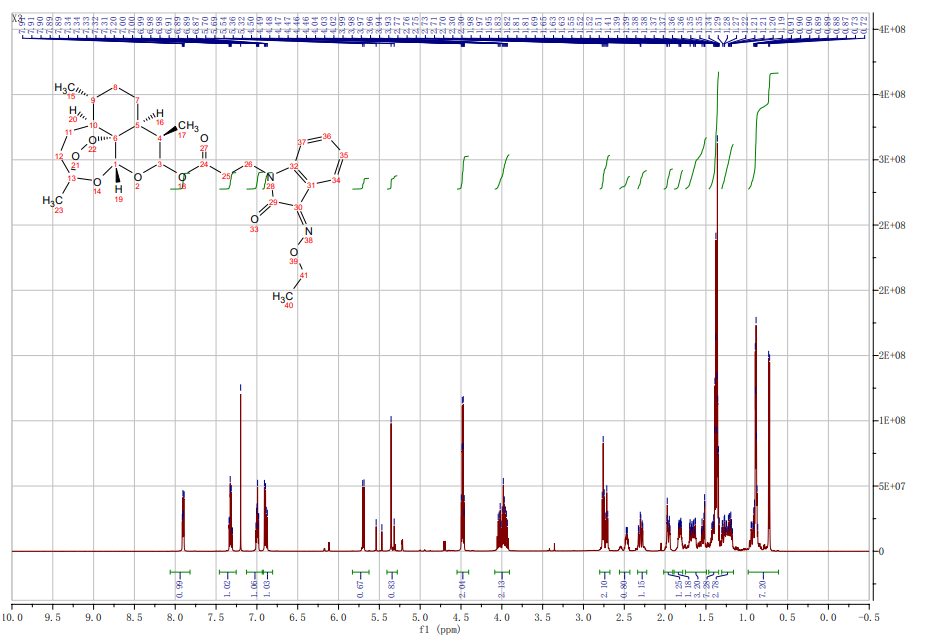


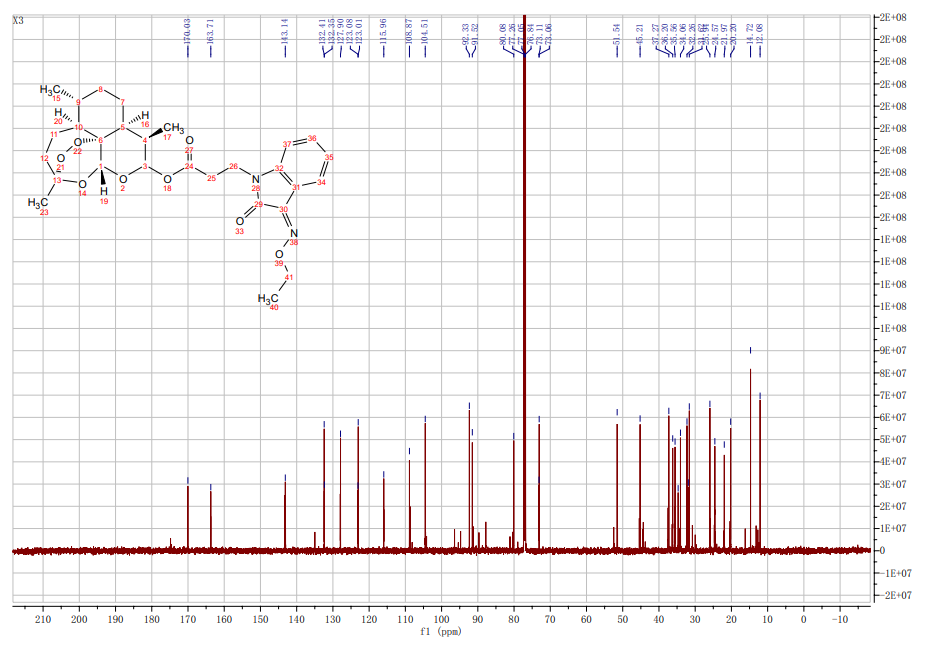


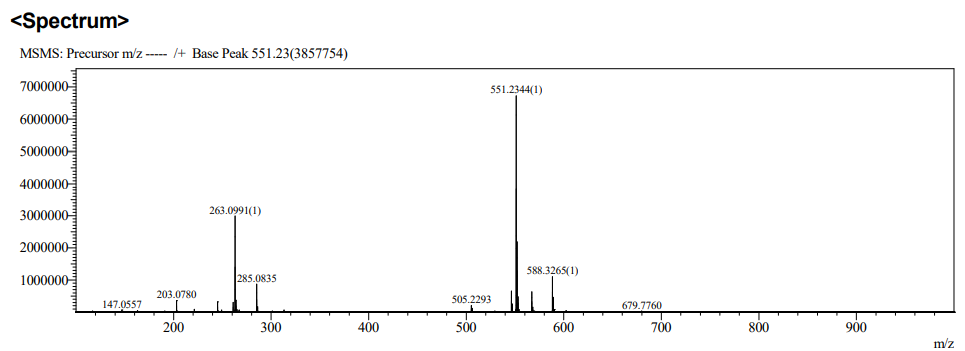


(3*R*,5a*S*,6*R*,8a*S*,9*R*,12*R*,12a*R*)- 3,6,9-trimethyldecahydro-12*H*-3,12-epoxy[1,2]dioxepino[4,3-*i*]isochromen-10-yl 3-(3-((benzyloxy)imino)-2-oxoindolin-1-yl)propanoate (**7g**)

^1^H NMR (600 MHz, CDCl_3_) δ 0.71-0.95 (m, 7H), 1.20-1.30 (m, 3H), 1.36-1.41 (m, 7H), 1.52-1.54 (m, 1H), 1.63-1.67 (m, 2H), 1.81-1.83 (m, 1H), 1.94-1.98 (m, 1H), 2.27-2.33 (m, 1H), 2.46-2.48 (m, 1H), 2.76 (t, *J* = 4.0 Hz, 2H), 3.92-4.04 (m, 2H), 5.36 (s, 1H), 5.46 (s, 1H), 5.70 (d, *J* = 4.0 Hz, 1H), 6.88 (d, *J* = 8.0 Hz, 1H), 6.96 (t, *J* = 8.0 Hz, 1H), 7.28-7.38 (m, 6H), 7.86 (d, *J* = 8.0 Hz, 1H). ^13^C NMR (150 MHz, CDCl_3_) 170.00, 163.67, 143.79, 143.29, 136.23, 132.64, 128.62, 128.47, 128.44, 128.16, 123.11, 116.90, 108.91, 104.61, 92.34, 91.82, 80.08, 79.41, 61.61, 46.21, 37.28, 36.21, 35.68, 34.07, 32.25, 31.63, 26.96, 24.38, 21.98, 20.21, 12.09. HRMS-ESI: m/z Calcd for C_33_H_38_N_2_O_8_Na [M+Na]^+^: 613.2520; Found: 613.2462.


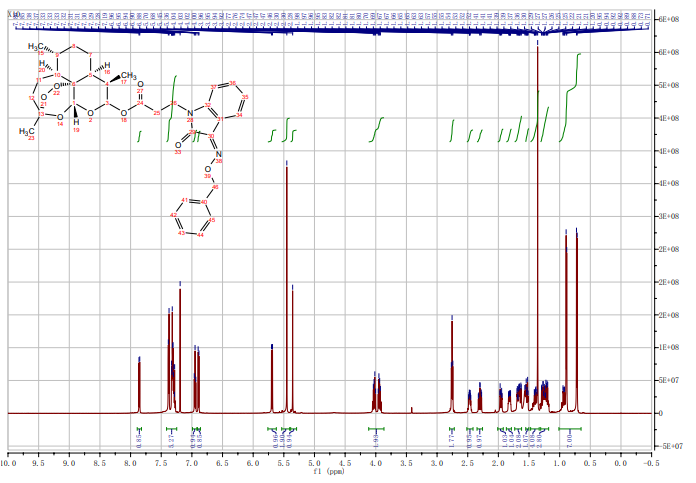


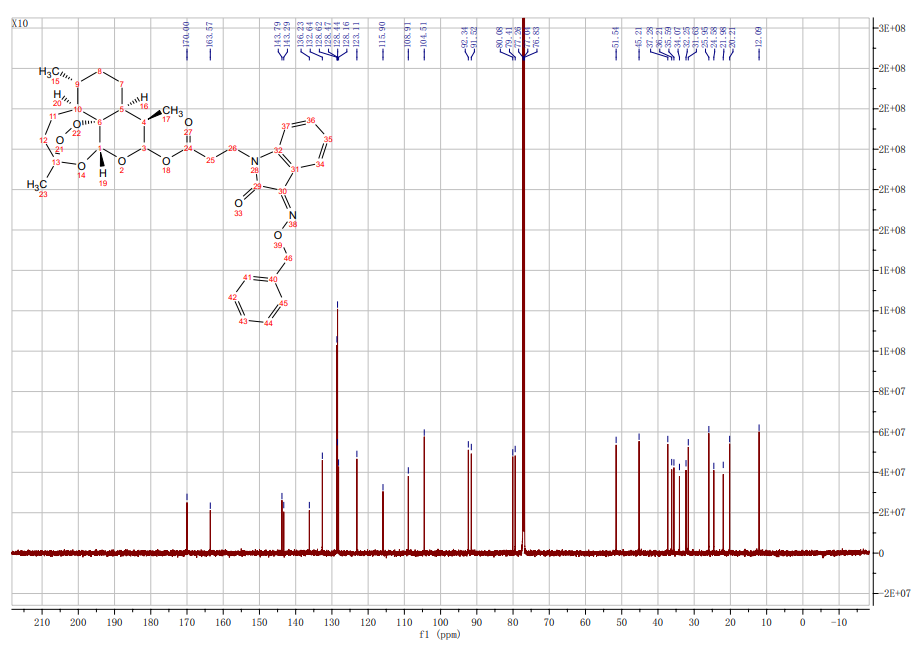


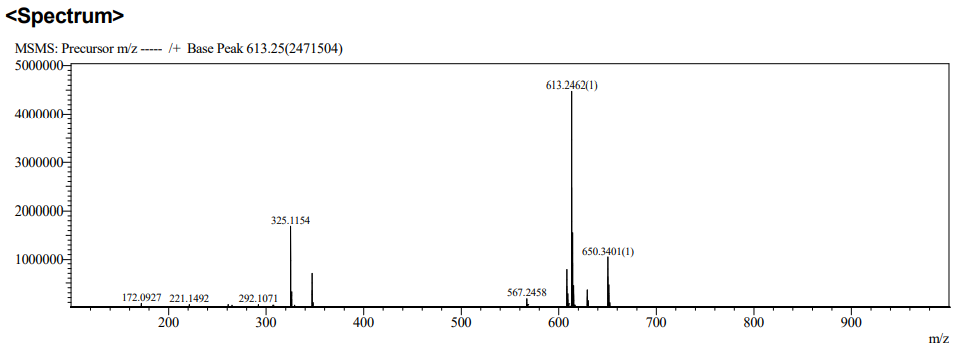


(3*R*,5a*S*,6*R*,8a*S*,9*R*,12*R*,12a*R*)-3,6,9-trimethyldecahydro-12*H*-3,12-epoxy[1,2]dioxepino[4,3-*i*]isochromen-10-yl 3-(5-fluoro-3-(methoxyimino)-2-oxoindolin-1-yl)propanoate (**7h**)

^1^H NMR (600 MHz, CDCl_3_) δ 0.72-0.96 (m, 7H), 1.20-1.41 (m, 8H), 1.53-1.56 (m, 1H), 1.63-1.70 (m, 2H), 1.81-1.83 (m, 1H), 1.94-1.98 (m, 1H), 2.28-2.33 (m, 1H), 2.42-2.48 (m, 1H), 3.74 (t, *J* = 4.0 Hz, 1H), 3.92-4.03 (m, 2H), 4.24 (s, 3H, NOMe), 5.36 (s, 1H), 5.68 (d, *J* = 4.0 Hz, 1H), 6.88 (dd, *J* = 4.0, 2.0 Hz, 1H), 7.06 (td, *J* = 8.0, 2.0 Hz, 1H), 7.62 (dd, *J* = 8.0, 2.0 Hz, 1H). ^13^C NMR (150 MHz, CDCl_3_) 170.04, 163.37, 159.83, 158.03, 143.03, 139.36, 118.91, 118.75, 116.30, 116.24, 115.61, 115.34, 109.80, 109.75, 104.61, 92.38, 91.61, 80.06, 65.06, 51.62, 46.19, 37.28, 36.19, 35.84, 34.06, 32.35, 31.61, 25.99, 24.57, 21.97, 20.20, 12.07. HRMS-ESI: m/z Calcd for C_27_H_33_FN_2_O_8_Na [M+Na]^+^: 555.2113; Found: 555.2077.


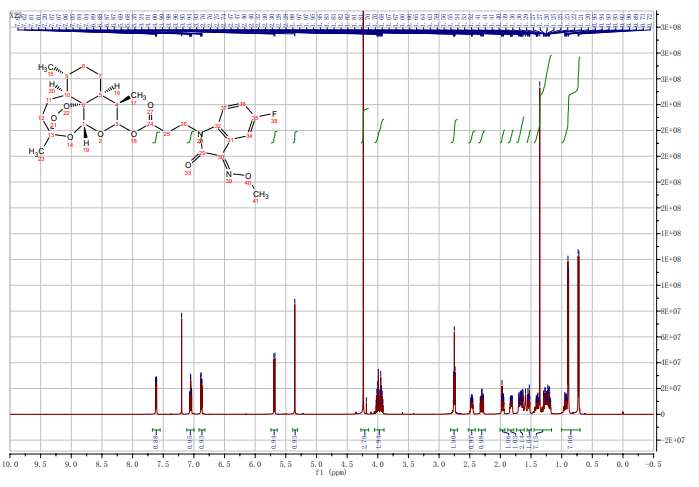


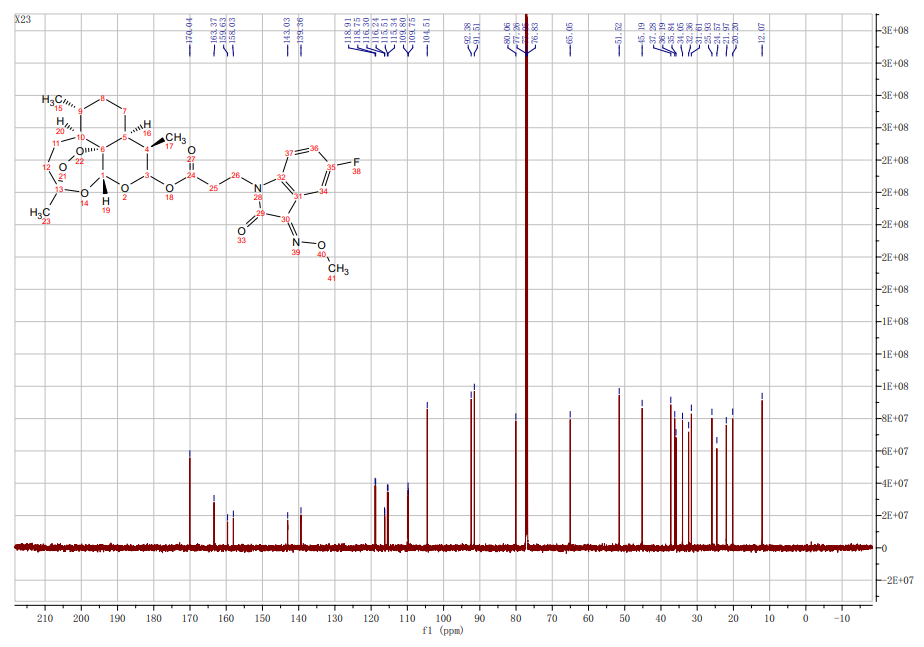


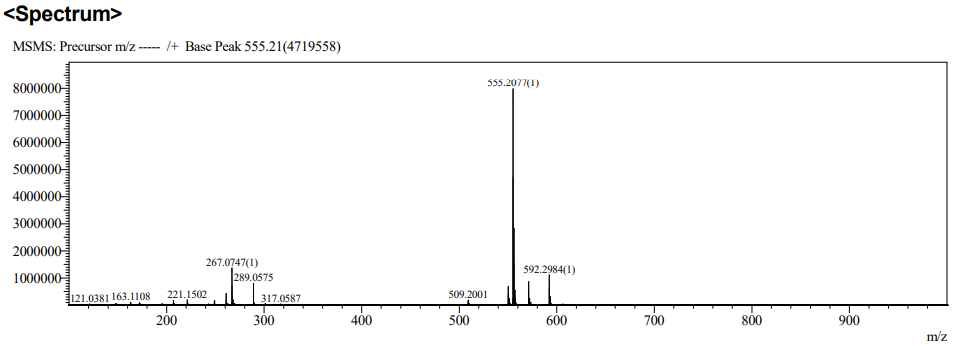


(3*R*,5a*S*,6*R*,8a*S*,9*R*,12*R*,12a*R*)-3,6,9-trimethyldecahydro-12*H*-3,12-epoxy[1,2]dioxepino[4,3-*i*]isochromen-10-yl 3-(3-(ethoxyimino)-5-fluoro-2-oxoindolin-1-yl)propanoate (**7i**)

^1^H NMR (600 MHz, CDCl_3_) δ 0.72-0.95 (m, 7H), 1.20-1.29 (m, 3H), 1.36-1.41 (m, 7H), 1.53-1.56 (m, 1H), 1.63-1.66 (m, 2H), 1.81-1.83 (m, 1H), 1.94-1.98 (m, 1H), 2.28-2.31 (m, 1H), 2.46-2.47 (m, 1H), 2.75 (t, *J* = 4.0 Hz, 2H), 3.93-4.02 (m, 2H), 4.50 (q, *J* = 4.0 Hz, 2H), 5.36 (s, 1H), 5.68 (d, *J* = 8.0 Hz, 1H), 6.88 (dd, *J* = 8.0, 4.0 Hz, 1H), 7.06 (td, *J* = 8.0, 2.0 Hz, 1H), 7.64 (dd, *J* = 4.0, 2.0 Hz, 1H). ^13^C NMR (150 MHz, CDCl_3_) 170.06, 163.51, 159.63, 158.03, 142.89, 139.24, 118.71, 118.66, 116.42, 116.37, 115.41, 115.23, 109.71, 109.66, 104.62, 92.38, 91.61, 80.06, 73.42, 61.83, 46.20, 37.28, 36.19, 35.81, 34.06, 32.37, 31.61, 25.93, 24.67, 21.97, 20.20, 14.71, 12.07. HRMS-ESI: m/z Calcd for C_27_H_33_FN_2_O_8_Na [M+Na]^+^: 569.2270; Found: 569.2240.


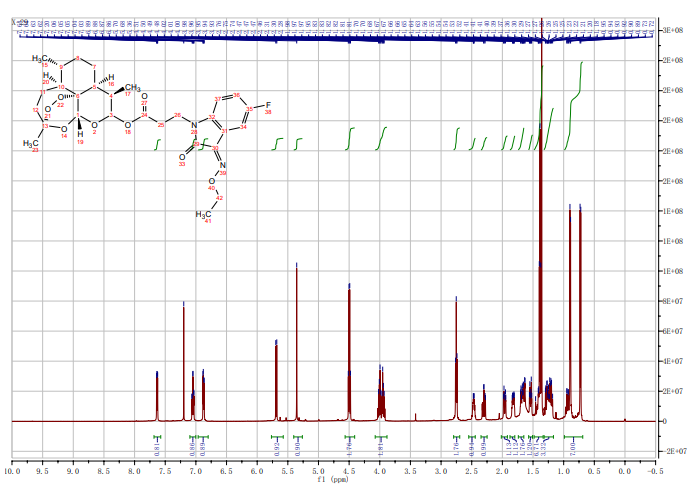


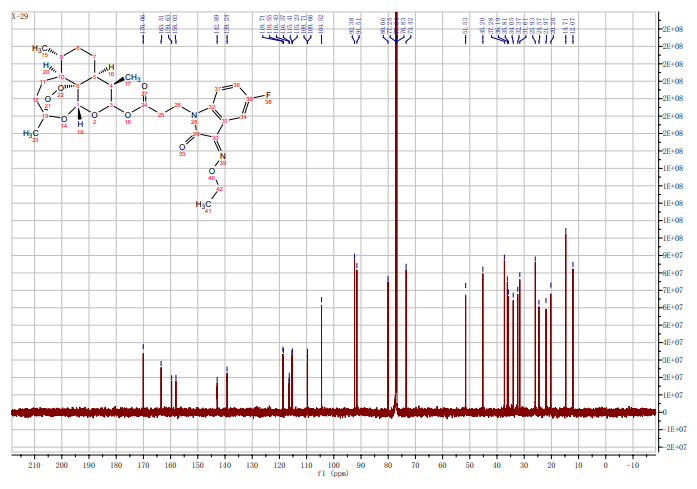


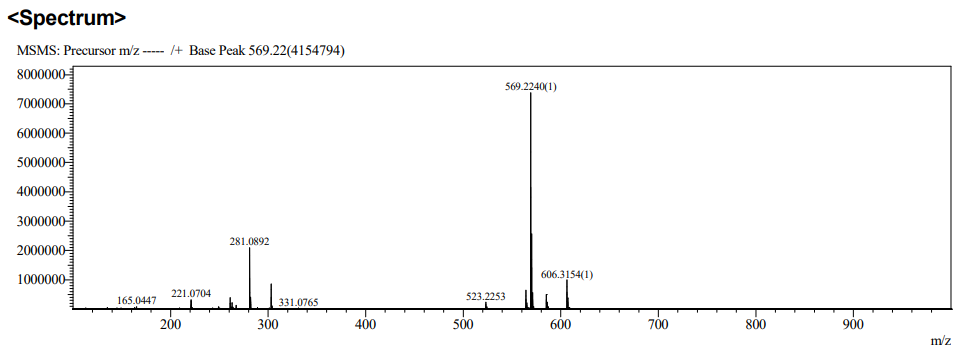


(3*R*,5a*S*,6*R*,8a*S*,9*R*,12*R*,12a*R*)-3,6,9-trimethyldecahydro-12*H*-3,12-epoxy[1,2]dioxepino[4,3-*i*]isochromen-10-yl 3-(3-((benzyloxy)imino)-5-fluoro-2-oxoindolin-1-yl)propanoate (**7j**)

^1^H NMR (600 MHz, CDCl_3_) δ 0.71-0.97 (m, 7H), 1.20-1.29 (m, 3H), 1.36-1.42 (m, 4H), 1.53-1.56 (m, 1H), 1.63-1.67 (m, 2H), 1.81-1.83 (m, 1H), 1.94-1.98 (m, 1H), 2.28-2.33 (m, 1H), 2.46-2.48 (m, 2H), 3.92-4.01 (m, 2H), 5.35 (s, 1H), 5.44 (s, 2H), 5.68 (d, *J* = 4.0 Hz, 1H), 6.86 (dd, *J* = 4.0, 2.0 Hz, 1H), 7.04 (td, *J* = 8.0, 2.0 Hz, 1H), 7.29-7.34 (m, 3H), 7.37-7.39 (m, 2H), 7.58 (dd, *J* = 8.0, 2.0 Hz, 1H). ^13^C NMR (150 MHz, CDCl_3_) 170.06, 163.39, 159.82, 158.02, 143.36, 139.39, 136.90, 128.70, 128.64, 128.63, 118.97, 118.81, 116.29, 115.61, 115.44, 109.80, 109.74, 104.82, 92.32, 91.62, 51.63, 46.20, 37.29, 36.20, 35.86, 34.06, 32.36, 31.61, 25.94, 24.67, 21.97, 20.20, 12.08. HRMS-ESI: m/z Calcd for C_33_H_37_FN_2_O_8_Na [M+Na]^+^: 631.2426; Found: 631.2393.


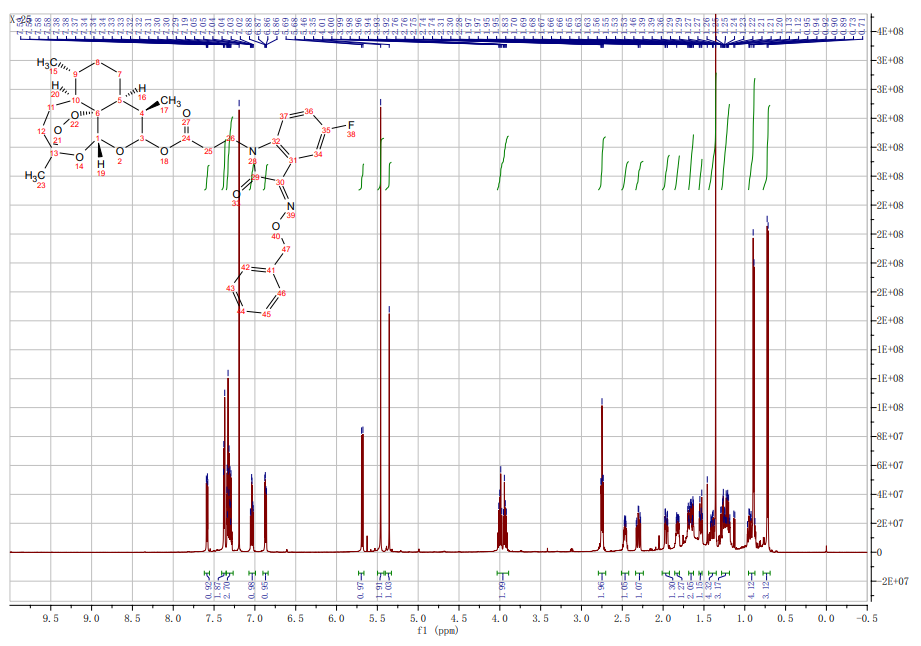


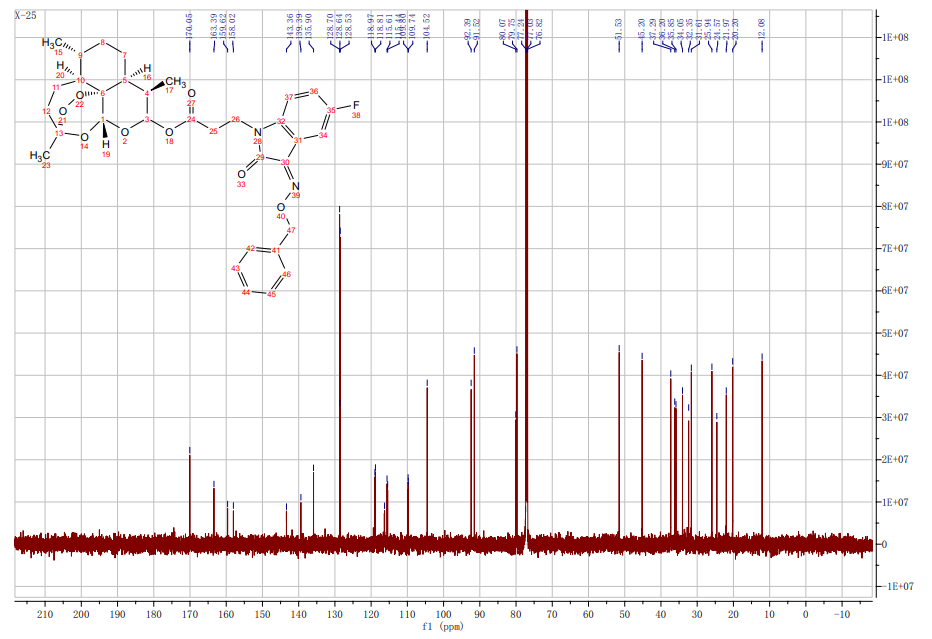


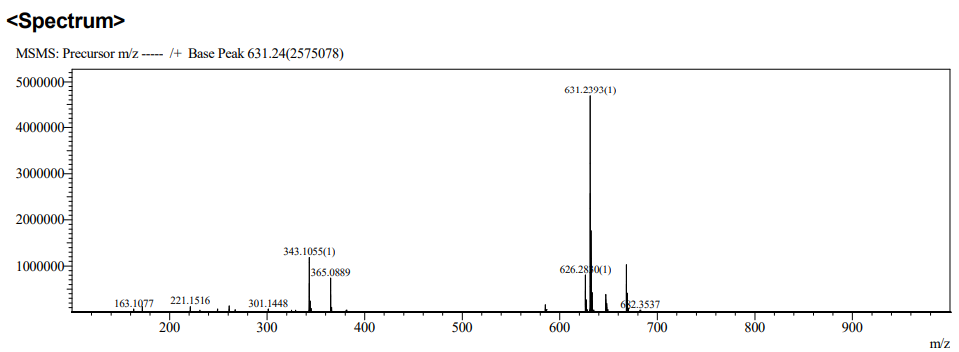


(3*R*,5a*S*,6*R*,8a*S*,9*R*,12*R*,12a*R*)-3,6,9-trimethyldecahydro-12*H*-3,12-epoxy[1,2]dioxepino[4,3-*i*]isochromen-10-yl 3-(5-methoxy-3-(methoxyimino)-2-oxoindolin-1-yl)propanoate (**7k**)

^1^H NMR (600 MHz, CDCl_3_) δ 0.72-0.95 (m, 7H), 1.18-1.43 (m, 7H), 1.51-1.53 (m, 1H), 1.63-1.70 (m, 2H), 1.80-1.84 (m, 1H), 1.94-1.98 (m, 1H), 2.27-2.33 (m, 1H), 2.46-2.49 (m, 1H), 2.68-2.76 (m, 2H), 3.74 (s, OMe), 3.89-4.01 (m, 2H), 4.22 (s, 3H, NOMe), 5.36 (s, 1H), 5.70 (d, *J* = 4.0 Hz, 1H), 6.78-6.82 (m, 1H), 6.85-6.88 (m, 1H), 7.40-7.42 (m, 1H). ^13^C NMR (150 MHz, CDCl_3_) 170.05, 163.45, 156.92, 156.87, 143.68, 136.98, 117.82, 117.43, 116.43, 116.40, 114.51, 114.50, 109.43, 109.25, 104.60, 92.33, 91.61, 80.08, 64.89, 61.64, 55.99, 46.21, 37.48, 37.37, 37.28, 36.20, 35.73, 35.67, 34.71, 34.06, 32.29, 31.90, 31.62, 26.02, 25.94, 24.67, 21.97, 20.20, 13.17, 12.08. HRMS-ESI: m/z Calcd for C_28_H_36_N_2_O_8_Na [M+Na]^+^: 567.2313; Found: 567.2275.


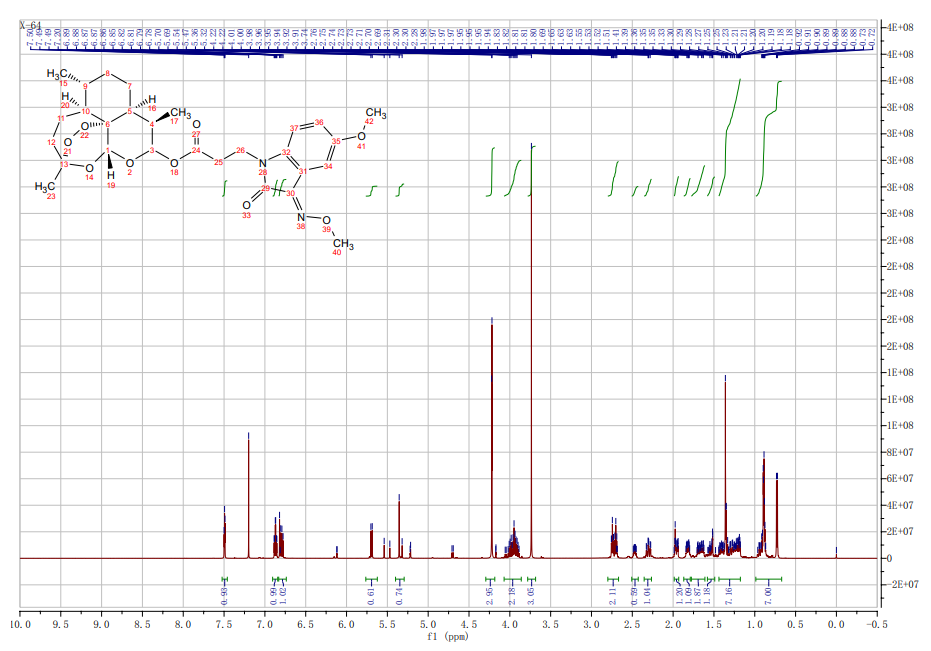


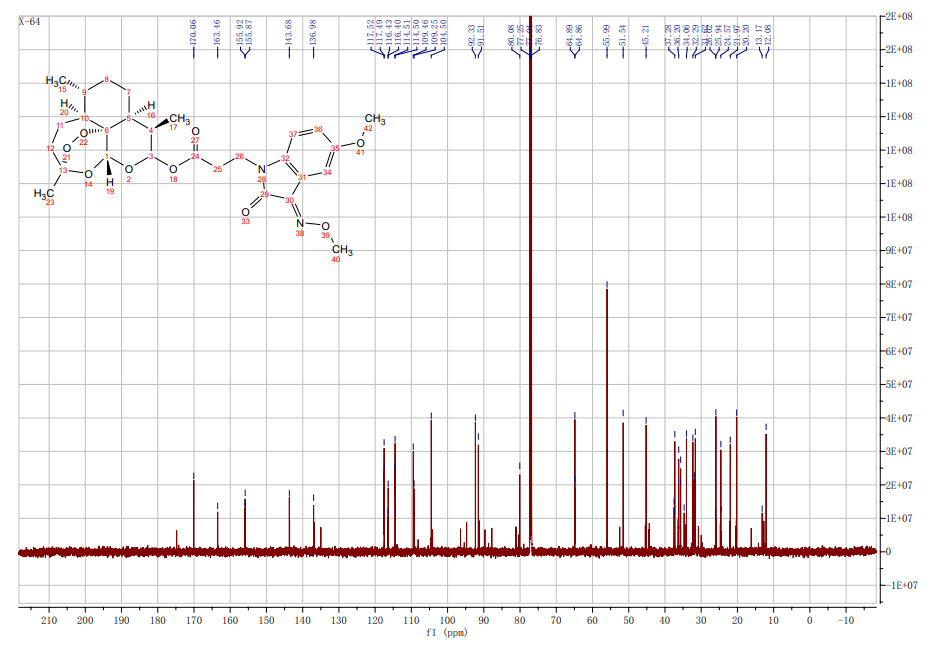


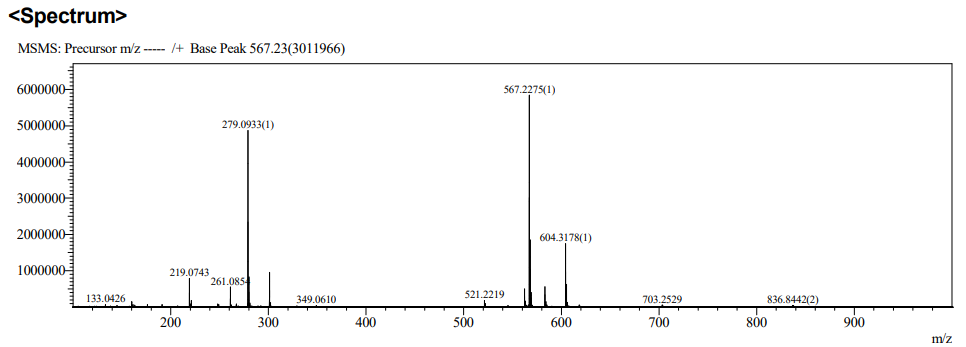


(3*R*,5a*S*,6*R*,8a*S*,9*R*,12*R*,12a*R*)-3,6,9-trimethyldecahydro-12*H*-3,12-epoxy[1,2]dioxepino[4,3-*i*]isochromen-10-yl 3-(5-methoxy-3-(ethoxyimino)-2-oxoindolin-1-yl)propanoate (**7l**)

^1^H NMR (600 MHz, CDCl_3_) δ 0.72-0.96 (m, 7H), 1.20-1.42 (m, 10H), 1.52-1.56 (m, 1H), 1.63-1.70 (m, 2H), 1.80-1.84 (m, 1H), 1.94-1.98 (m, 1H), 2.23-2.33 (m, 1H), 2.46-2.48 (m, 1H), 2.68-2.76 (m, 2H), 3.74 (s, 3H, OMe), 3.89-4.03 (m, 2H), 4.48 (q, *J* = 8.0 Hz, 2H), 5.36 (s, 1H), 5.70 (d, *J* = 4.0 Hz, 1H), 6.78-6.80 (m, 1H), 6.86-6.88 (m, 1H), 7.51-7.54 (m, 1H). ^13^C NMR (150 MHz, CDCl_3_) 170.08, 163.57, 156.83, 143.63, 136.87, 117.04, 116.86, 114.88, 109.33, 104.60, 92.31, 91.62, 80.08, 73.10, 55.94, 51.54, 45.21, 37.28, 36.21, 35.64, 34.06, 32.30, 31.63, 26.96, 24.67, 21.98, 20.21, 14.72, 12.09. HRMS-ESI: m/z Calcd for C_29_H_38_N_2_O_8_Na [M+Na]^+^: 581.2470; Found: 581.2442.


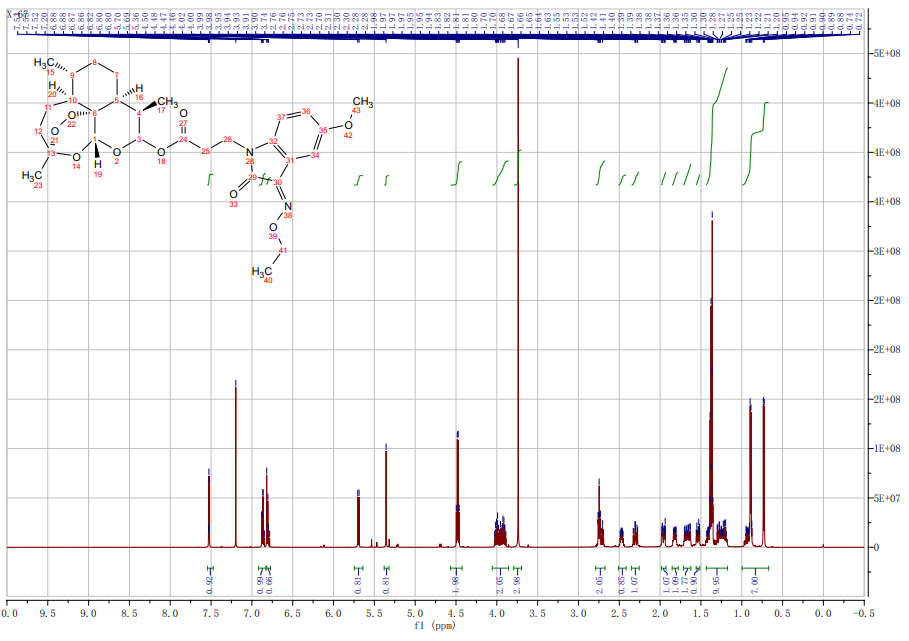


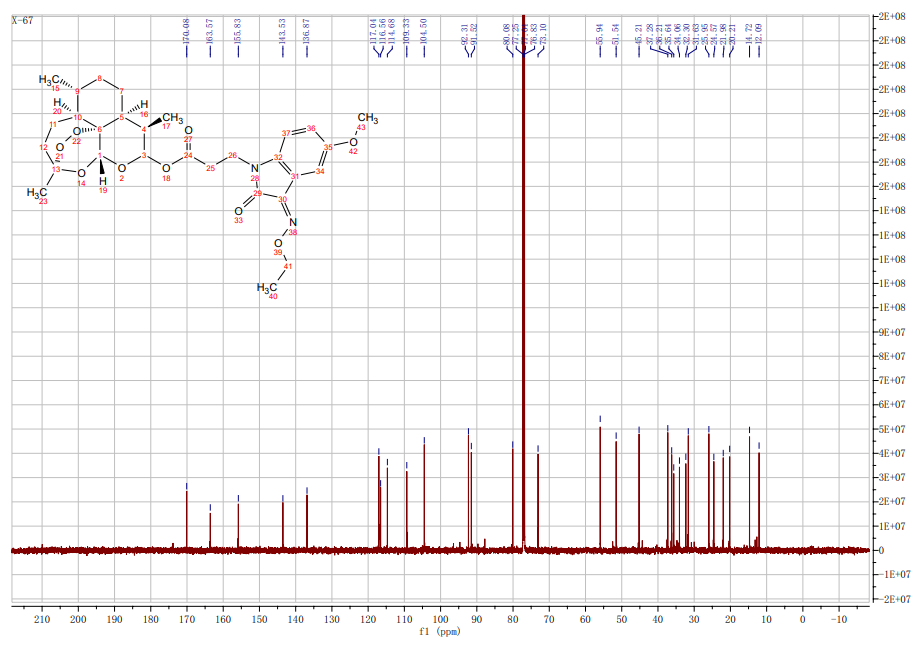


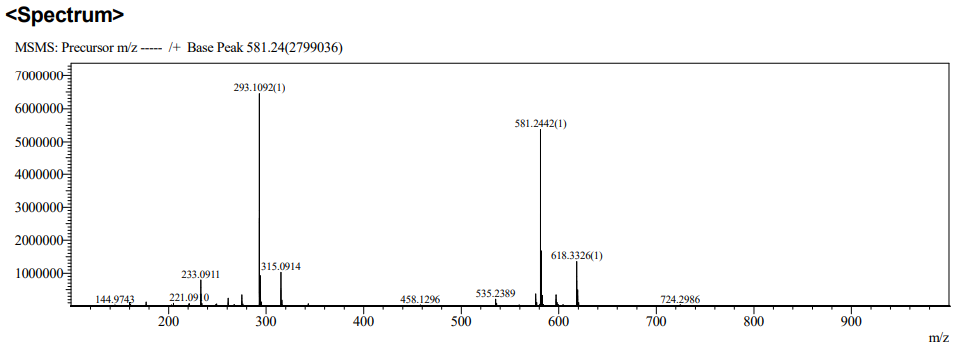


(3*R*,5a*S*,6*R*,8a*S*,9*R*,12*R*,12a*R*)-3,6,9-trimethyldecahydro-12*H*-3,12-epoxy[1,2]dioxepino[4,3-*i*]isochromen-10-yl 4-(3-(methoxyimino)-2-oxoindolin-1-yl)butanoate (**7m**)

^1^H NMR (600 MHz, CDCl_3_) δ 0.77-0.96 (m, 7H), 1.19-1.43 (m, 7H), 1.54-1.58 (m, 1H), 1.64-1.73 (m, 2H), 1.81-1.84 (m, 1H), 1.91-1.99 (m, 3H), 2.28-2.33 (m, 1H), 2.49-2.61 (m, 3H), 3.67-3.79 (m, 2H), 4.22 (s, 3H, NOMe), 5.39 (s, 1H), 5.73 (d, *J* = 4.0 Hz, 1H), 6.92 (d, *J* = 4.0 Hz, 1H), 6.98 (t, *J* = 4.0 Hz, 1H), 7.34 (t, *J* = 4.0 Hz, 1H), 7.88 (d, *J* = 4.0 Hz, 1H). ^13^C NMR (150 MHz, CDCl_3_) 171.71, 163.80, 143.68, 143.61, 132.77, 127.95, 122.93, 116.76, 108.97, 104.49, 92.13, 91.60, 80.10, 64.75, 61.86, 45.23, 38.94, 37.29, 36.22, 34.09, 31.71, 31.03, 26.96, 24.89, 22.36, 22.00, 20.22, 12.16. HRMS-ESI: m/z Calcd for C_28_H_36_N_2_O_8_Na [M+Na]^+^: 551.2364; Found: 551.2318.


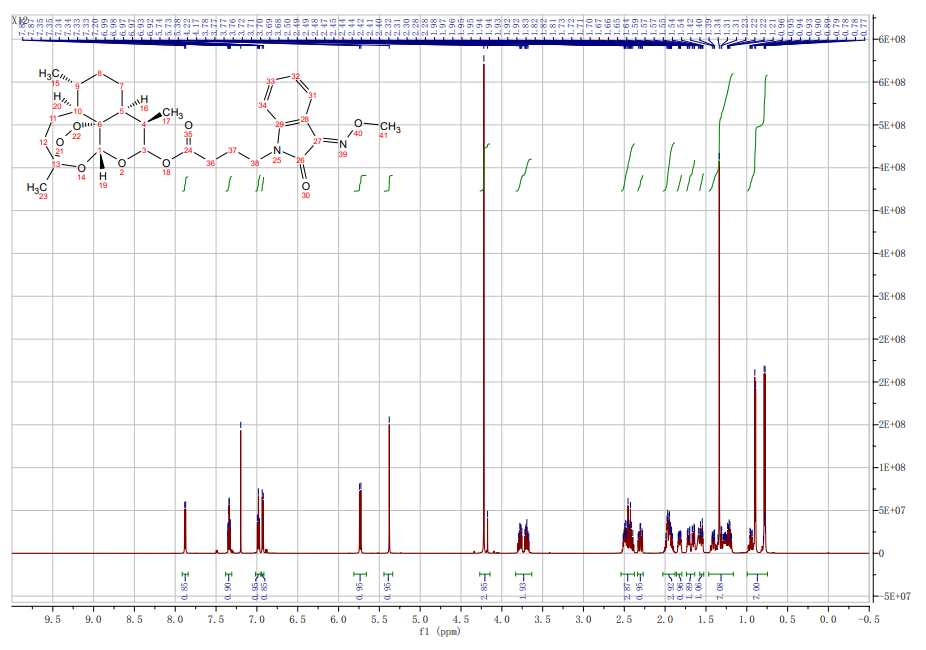


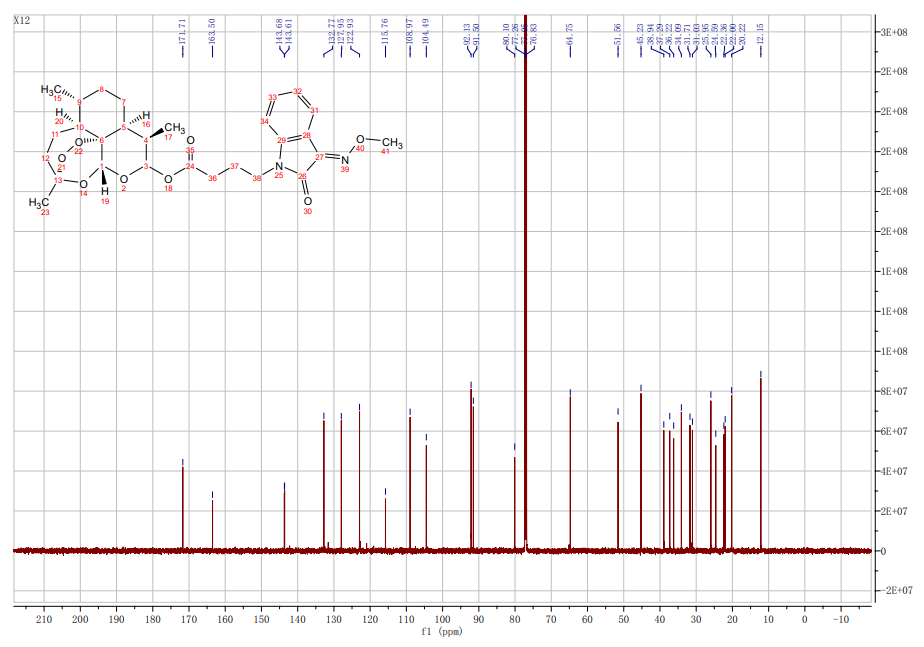


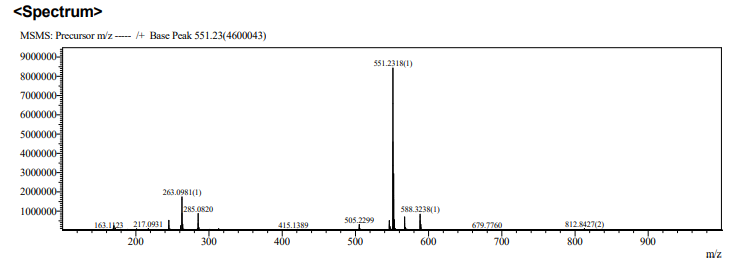


(3*R*,5a*S*,6*R*,8a*S*,9*R*,12*R*,12a*R*)-3,6,9-trimethyldecahydro-12*H*-3,12-epoxy[1,2]dioxepino[4,3-*i*]isochromen-10-yl 4-(3-(ethoxyimino)-2-oxoindolin-1-yl)butanoate (**7n**)

^1^H NMR (600 MHz, CDCl_3_) δ 0.77-0.96 (m, 7H), 1.19-1.27 (m, 2H), 1.31-1.42 (m, 8H), 1.52-1.57 (m, 1H), 1.64-1.72 (m, 2H), 1.81-1.83 (m, 1H), 1.91-1.99 (m, 3H), 2.28-2.33 (m, 1H), 2.40-2.52 (m, 3H), 3.70-3.80 (m, 2H), 4.48 (q, *J* = 4.0 Hz, 1H), 5.38 (s, 1H), 5.74 (d, *J* = 4.0 Hz, 1H), 6.92 (d, *J* = 4.0 Hz, 1H), 6.98 (t, *J* = 4.0 Hz, 1H), 7.34 (t, *J* = 8.0 Hz, 1H), 7.90 (d, *J* = 8.0 Hz, 1H). ^13^C NMR (150 MHz, CDCl_3_) 171.73, 163.64, 143.86, 143.64, 132.68, 127.85, 122.89, 116.69, 108.90, 104.49, 92.13, 91.60, 80.10, 72.99, 61.60, 45.23, 38.91, 37.29, 36.22, 34.09, 31.71, 31.06, 26.94, 24.89, 22.38, 22.00, 20.22, 14.73, 12.15. HRMS-ESI: m/z Calcd for C_29_H_38_N_2_O_8_Na [M+Na]^+^: 565.2520; Found: 565.2484.


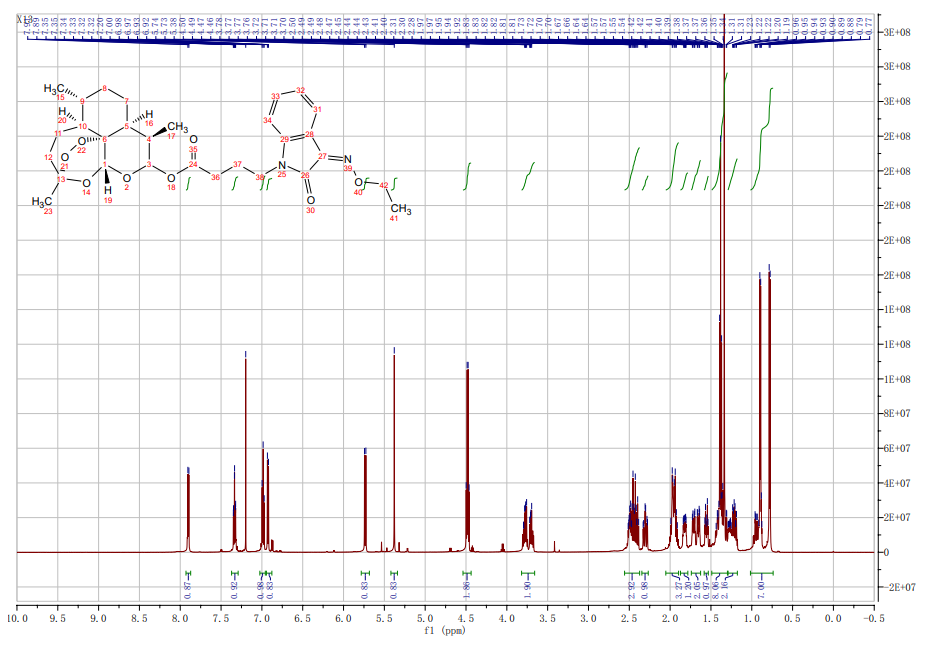


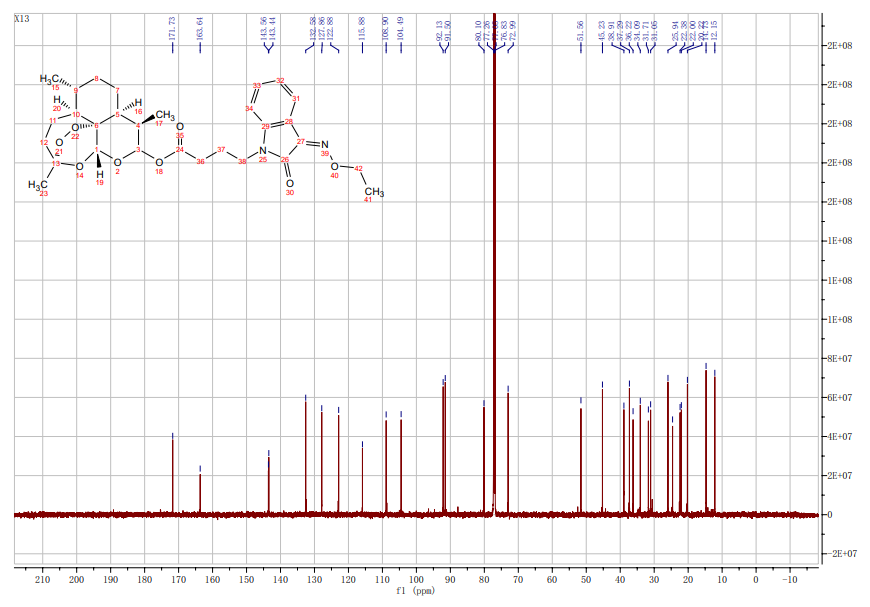


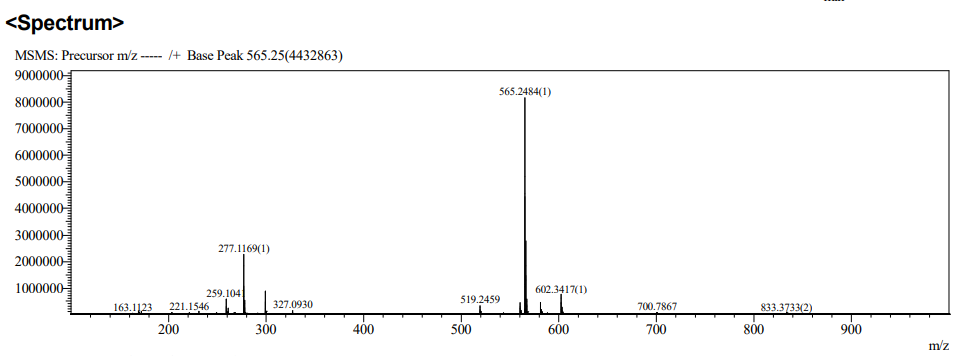


(3*R*,5a*S*,6*R*,8a*S*,9*R*,12*R*,12a*R*)-3,6,9-trimethyldecahydro-12*H*-3,12-epoxy[1,2]dioxepino[4,3-*i*]isochromen-10-yl 4-(3-((benzyloxy)imino)-2-oxoindolin-1-yl)butanoate (**70**)

^1^H NMR (600 MHz, CDCl_3_) δ 0.77-0.96 (m, 7H), 1.18-1.42 (m, 7H), 1.53-1.57 (m, 1H), 1.64-1.72 (m, 2H), 1.80-1.83 (m, 1H), 1.90-1.98 (m, 3H), 2.27-2.33 (m, 1H), 2.39-2.52 (m, 3H), 3.65-3.78 (m, 2H), 5.37 (s, 1H), 5.46 (s, 2H), 5.72 (d, *J* = 8.0 Hz, 1H), 6.91-6.95 (m, 2H), 7.28-7.33 (m, 4H), 7.37-7.39 (m, 2H), 7.86 (d, *J* = 4.0 Hz, 1H). ^13^C NMR (150 MHz, CDCl_3_) 171.72, 163.63, 143.98, 143.69, 136.27, 132.82, 128.52, 128.46, 128.44, 128.35, 128.19, 128.12, 122.99, 116.81, 108.96, 104.50, 92.16, 91.61, 80.11, 79.36, 61.67, 45.23, 38.96, 37.29, 36.22, 34.09, 31.71, 31.04, 25.95, 24.89, 22.36, 22.01, 20.22, 12.16. HRMS-ESI: m/z Calcd for C_34_H_40_N_2_O_8_Na [M+Na]^+^: 627.2647; Found: 627.2617.


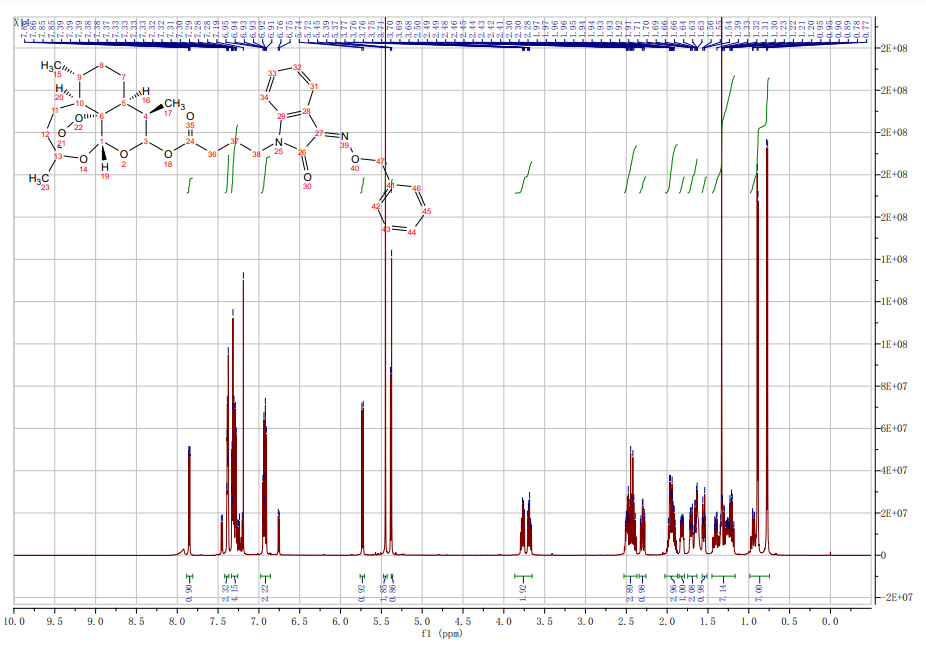


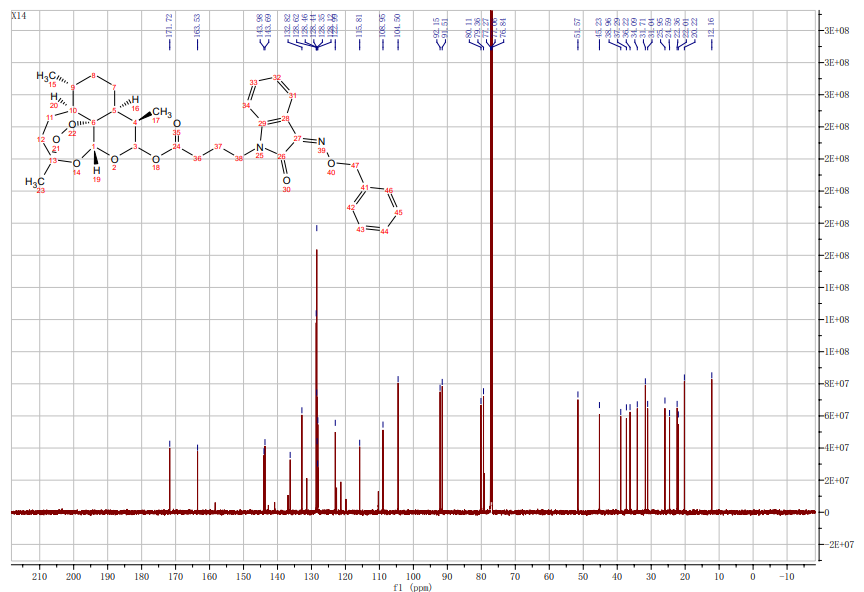


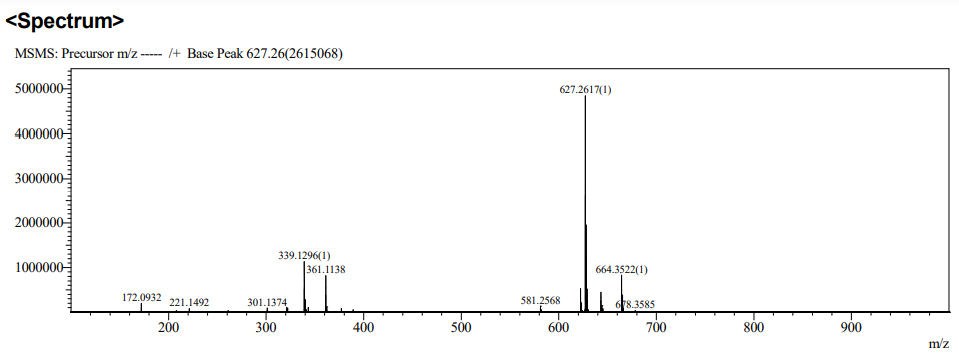


(3*R*,5a*S*,6*R*,8a*S*,9*R*,12*R*,12a*R*)-3,6,9-trimethyldecahydro-12*H*-3,12-epoxy[1,2]dioxepino[4,3-*i*]isochromen-10-yl 4-(3-(methoxyimino)-5-fluoro-2-oxoindolin-1-yl)butanoate (**7p**)

^1^H NMR (600 MHz, CDCl_3_) δ 0.77-0.96 (m, 7H), 1.20-1.42 (m, 7H), 1.53-1.57 (m, 1H), 1.64-1.72 (m, 2H), 1.80-1.84 (m, 1H), 1.91-1.98 (m, 3H), 2.26-2.33 (m, 1H), 2.37-2.50 (m, 3H), 3.66-3.77 (m, 2H), 4.22 (s, 3H, NOMe), 5.37 (s, 1H), 5.74 (d, *J* = 8.0 Hz, 1H), 6.80 (d, *J* = 4.0 Hz, 1H), 7.14 (d, *J* = 4.0 Hz, 1H), 7.71 (s, 1H). ^13^C NMR (150 MHz, CDCl_3_) 171.73, 163.66, 143.80, 141.43, 133.03, 132.49, 128.09, 116.78, 108.71, 104.49, 92.11, 91.80, 80.10, 64.89, 51.57, 46.23, 38.97, 37.29, 36.22, 34.09, 31.71, 31.05, 25.94, 24.68, 22.38, 22.00, 20.96, 20.22, 12.14. HRMS-ESI: m/z Calcd for C_28_H_38_FN_2_O_9_ [M+H_3_O]^+^: 565.2455; Found: 565.2496.


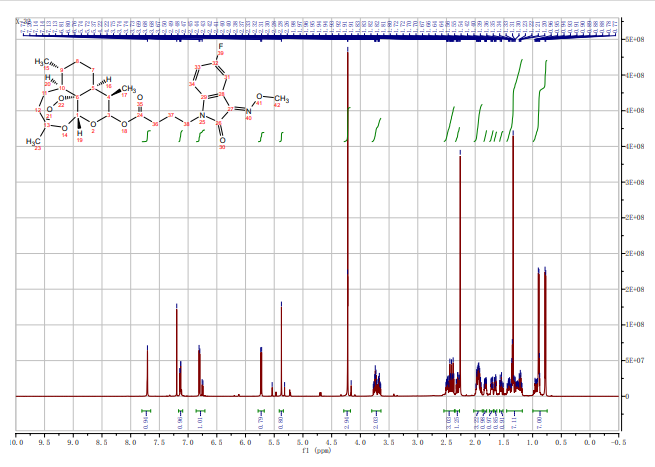


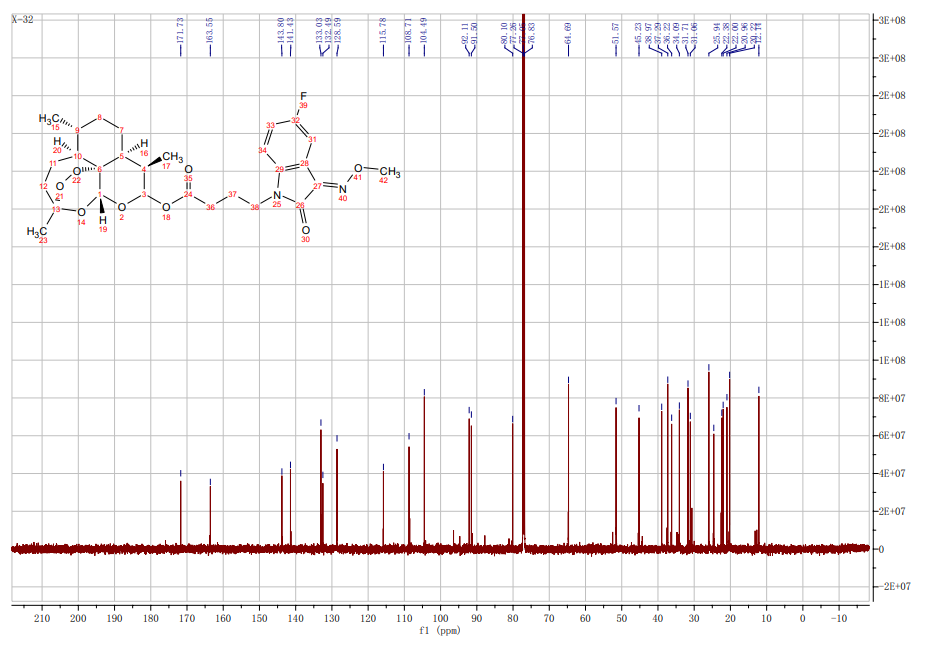


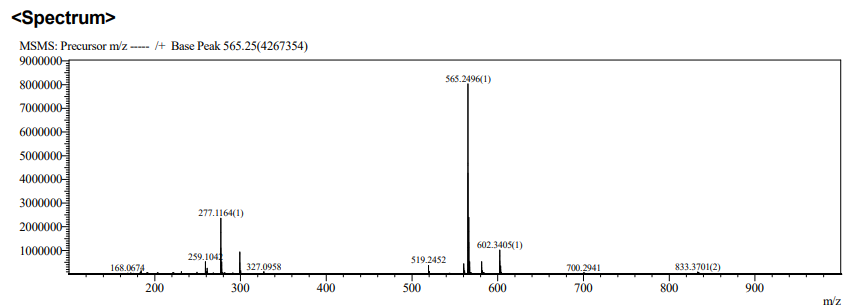


(3*R*,5a*S*,6*R*,8a*S*,9*R*,12*R*,12a*R*)-3,6,9-trimethyldecahydro-12*H*-3,12-epoxy[1,2]dioxepino[4,3-*i*]isochromen-10-yl 4-(3-(ethoxyimino)-5-fluoro-2-oxoindolin-1-yl)butanoate (**7q**)

^1^H NMR (600 MHz, CDCl_3_) δ 0.77-0.96 (m, 7H), 1.19-1.44 (m, 10H), 1.56-1.58 (m, 1H), 1.64-1.73 (m, 2H), 1.81-1.84 (m, 1H), 1.90-1.97 (m, 3H), 2.28-2.33 (m, 1H), 2.39-2.51 (m, 3H), 3.65-3.79 (m, 2H), 4.60 (q, *J* = 4.0 Hz, 2H), 5.38 (s, 1H), 5.74 (d, *J* = 4.0 Hz, 1H), 6.80-6.91 (m, 1H), 7.03-7.09 (m, 1H), 7.62-7.66 (m, 1H). ^13^C NMR (150 MHz, CDCl_3_) 176.29, 171.72, 163.42, 159.83, 158.04, 143.09, 139.60, 118.91, 118.76, 118.69, 118.53, 116.36, 116.24, 116.03, 115.42, 115.25, 109.64, 109.50, 109.30, 104.32, 92.18, 91.82, 80.10, 73.44, 73.36, 51.86, 45.22, 39.07, 39.04, 37.49, 37.30, 36.35, 36.21, 34.21, 34.08, 31.71, 30.92, 30.62, 25.93, 24.89, 22.83, 22.26, 22.00, 20.22, 14.71, 13.18, 12.14. HRMS-ESI: m/z Calcd for C_29_H_37_FN_2_O_8_Na [M+Na]^+^: 583.2426; Found: 583.2405.


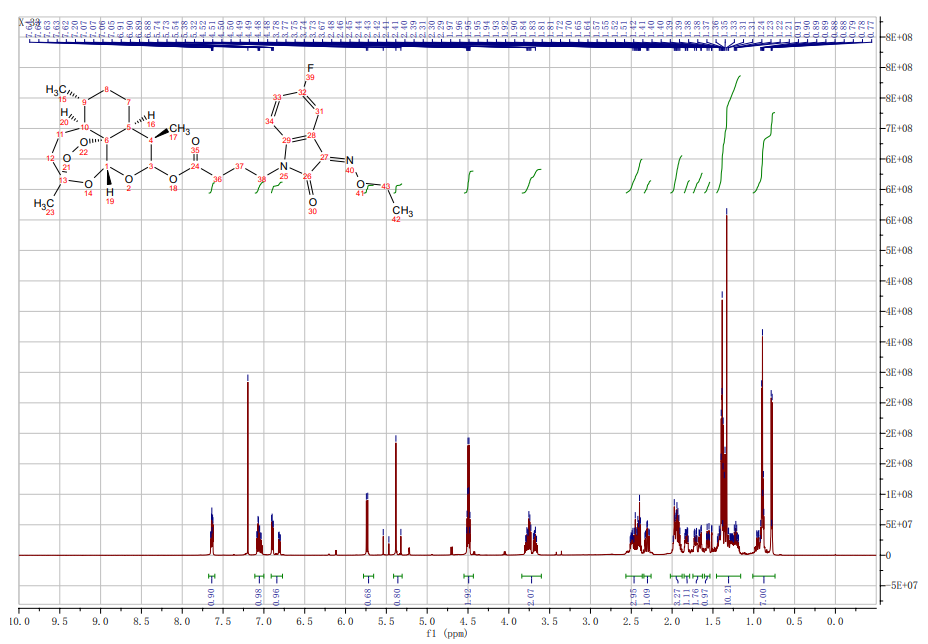


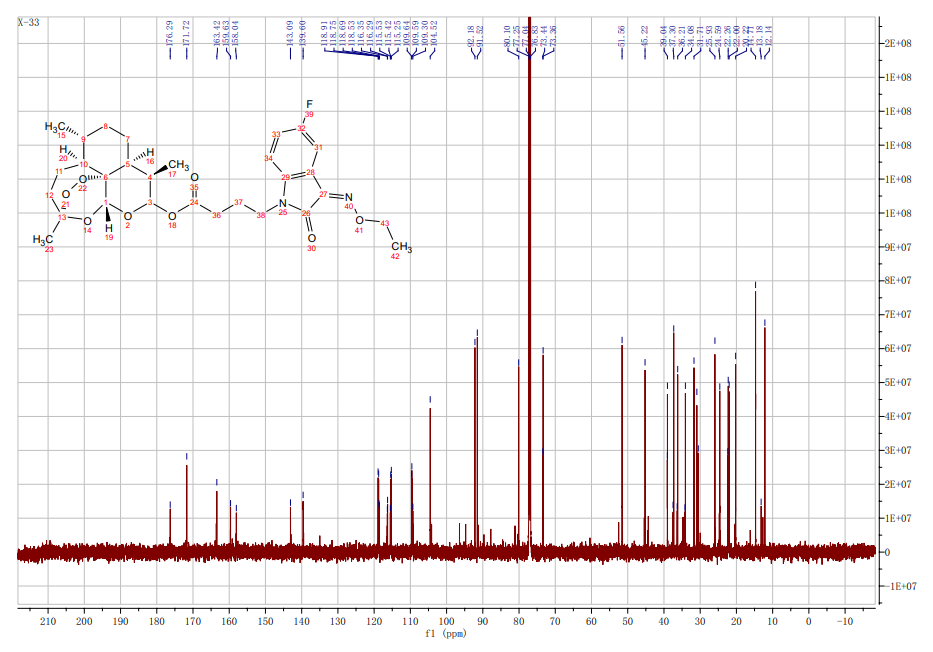


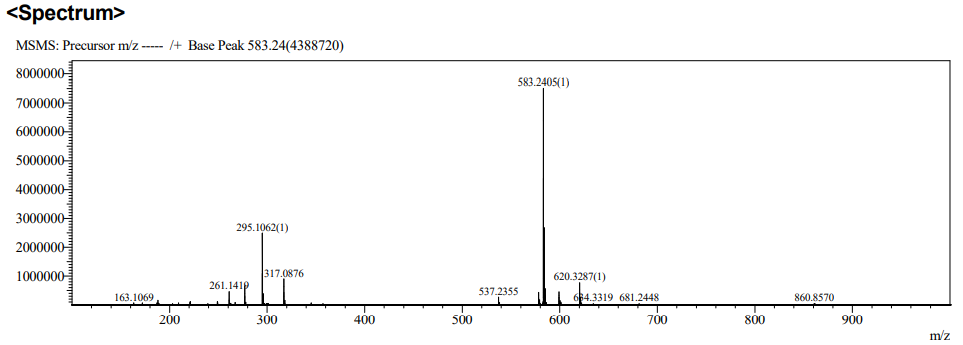


(3*R*,5a*S*,6*R*,8a*S*,9*R*,12*R*,12a*R*)-3,6,9-trimethyldecahydro-12*H*-3,12-epoxy[1,2]dioxepino[4,3-*i*]isochromen-10-yl 4-(3-((benzyloxy)imino)-5-fluoro-2-oxoindolin-1-yl)butanoate (**7r**)

^1^H NMR (600 MHz, CDCl_3_) δ 0.77-0.96 (m, 7H), 1.21-1.42 (m, 7H), 1.54-1.58 (m, 1H), 1.64-1.72 (m, 2H), 1.81-1.85 (m, 1H), 1.90-1.98 (m, 3H), 2.28-2.33 (m, 1H), 2.39-2.52 (m, 3H), 3.64-3.80 (m, 2H), 5.38 (s, 1H), 5.46 (s, 2H), 5.74 (d, *J* = 4.0 Hz, 1H), 6.88 (dd, *J* = 8.0, 4.0 Hz, 1H), 7.06 (td, *J* = 8.0, 2.0 Hz, 1H), 7.58 (dd, *J* = 8.0, 2.0 Hz, 1H). ^13^C NMR (150 MHz, CDCl_3_) 171.84, 163.27, 159.61, 158.02, 143.68, 139.73, 136.95, 128.69, 128.62, 128.52, 119.18, 119.02, 116.27, 116.21, 115.62, 115.45, 109.71, 109.66, 104.62, 92.18, 91.82, 80.10, 79.89, 51.66, 45.22, 39.07, 37.30, 36.21, 34.09, 31.71, 30.92, 26.94, 24.69, 22.25, 22.01, 20.22, 12.16. HRMS-ESI: m/z Calcd for C_34_H_39_FN_2_O_8_Na [M+Na]^+^: 645.2583; Found: 645.2561.


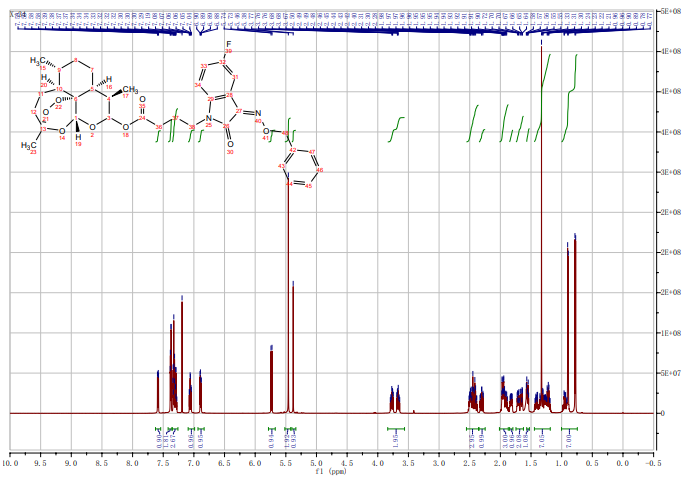


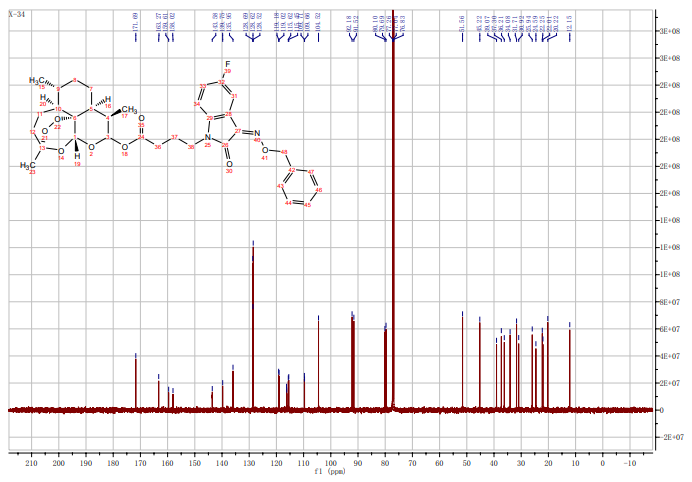


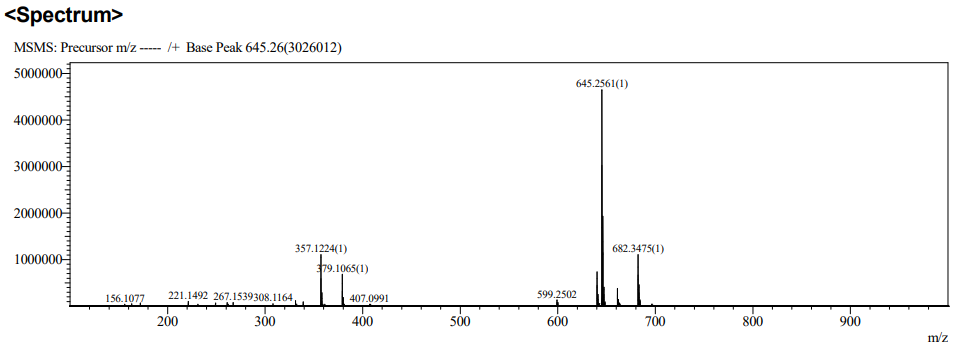


(3*R*,5a*S*,6*R*,8a*S*,9*R*,12*R*,12a*R*)-3,6,9-trimethyldecahydro-12*H*-3,12-epoxy[1,2]dioxepino[4,3-*i*]isochromen-10-yl 4-(5-methoxy-3-(methoxyimino)-2-oxoindolin-1-yl)butanoate (**7s**)

^1^H NMR (600 MHz, CDCl_3_) δ 0.77-0.96 (m, 7H), 1.19-1.42 (m, 7H), 1.54-1.57 (m, 1H), 1.64-1.73 (m, 2H), 1.81-1.85 (m, 1H), 1.90-1.99 (m, 3H), 2.28-2.33 (m, 1H), 2.37-2.52 (m, 3H), 3.63-3.77 (m, 5H), 4.42 (s, 3H, NOMe), 5.38 (s, 1H), 5.74 (d, *J* = 8.0 Hz, 1H), 6.79-6.91 (m, 2H), 7.50 (d, *J* = 2.0 Hz, 1H). ^13^C NMR (150 MHz, CDCl_3_) 171.72, 163.36, 156.82, 143.89, 137.41, 117.77, 116.31, 114.45, 109.47, 104.49, 92.13, 91.80, 80.09, 64.80, 56.00, 51.57, 45.23, 38.99, 37.29, 36.22, 34.09, 31.71, 31.03, 25.95, 24.69, 22.35, 22.00, 20.22, 12.15. HRMS-ESI: m/z Calcd for C_29_H_38_N_2_O_9_Na [M+Na]^+^: 581.2470; Found: 581.2440.


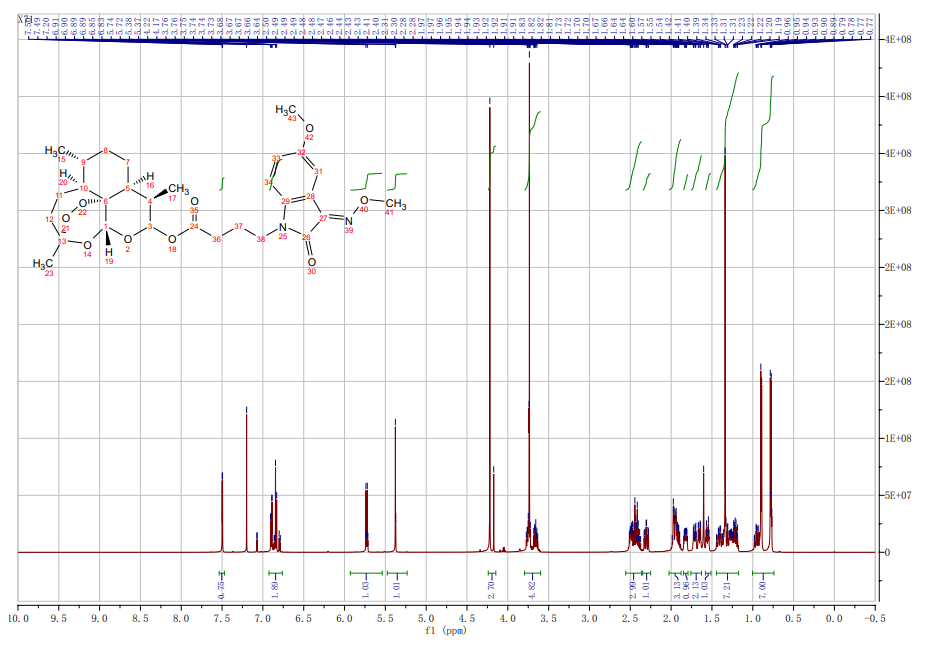


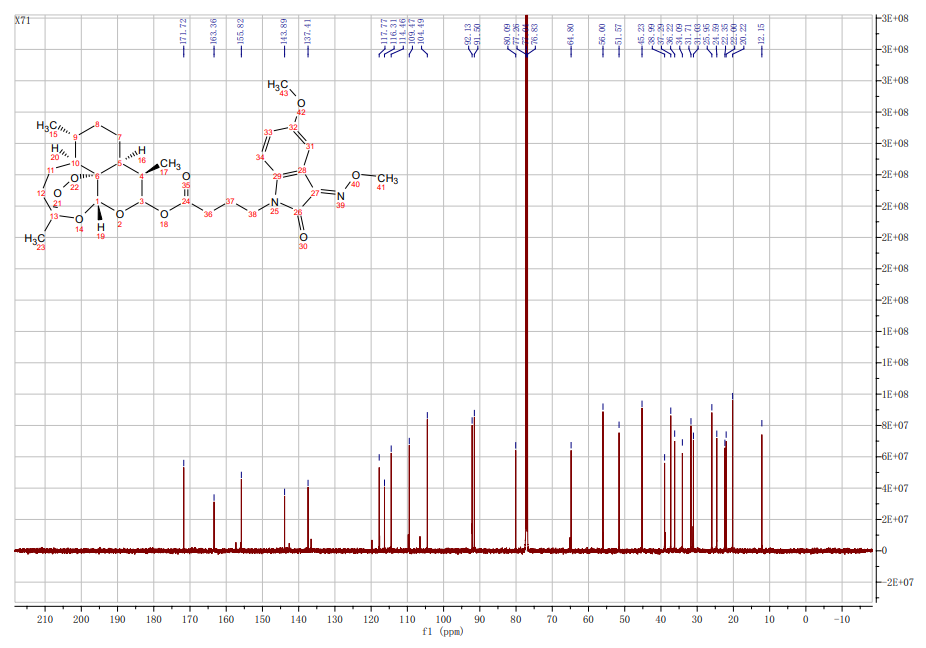


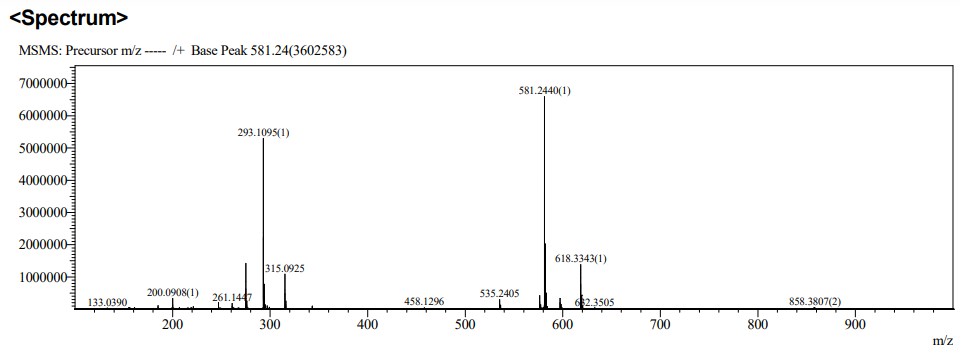


(3*R*,5a*S*,6*R*,8a*S*,9*R*,12*R*,12a*R*)-3,6,9-trimethyldecahydro-12*H*-3,12-epoxy[1,2]dioxepino[4,3-*i*]isochromen-10-yl 4-(3-(ethoxyimino)-5-methoxy-2-oxoindolin-1-yl)butanoate (**7t**)

^1^H NMR (600 MHz, CDCl_3_) δ 0.77-0.96 (m, 7H), 1.21-1.43 (m, 10H), 1.52-1.57 (m, 1H), 1.64-1.73 (m, 2H), 1.81-1.84 (m, 1H), 1.91-1.98 (m, 3H), 2.28-2.33 (m, 1H), 2.38-2.52 (m, 3H), 3.64-3.78 (m, 5H), 4.48 (q, *J* = 8.0 Hz, 2H), 5.38 (s, 1H), 5.74 (d, *J* = 8.0 Hz, 1H), 6.76-6.90 (m, 2H), 7.53 (d, *J* = 2.0 Hz, 1H). ^13^C NMR (150 MHz, CDCl_3_) 171.75, 163.30, 156.78, 143.72, 137.29, 117.29 116.47, 114.65, 109.38, 104.30, 92.12, 91.61, 80.10, 73.04, 55.95, 51.57, 45.23, 38.97, 37.29, 36.22, 34.09, 31.72, 31.05, 25.95, 22.35, 22.01, 20.22, 14.74, 12.15. HRMS-ESI: m/z Calcd for C_30_H_40_N_2_O_9_Na [M+Na]^+^: 595.2626; Found: 595.2605.


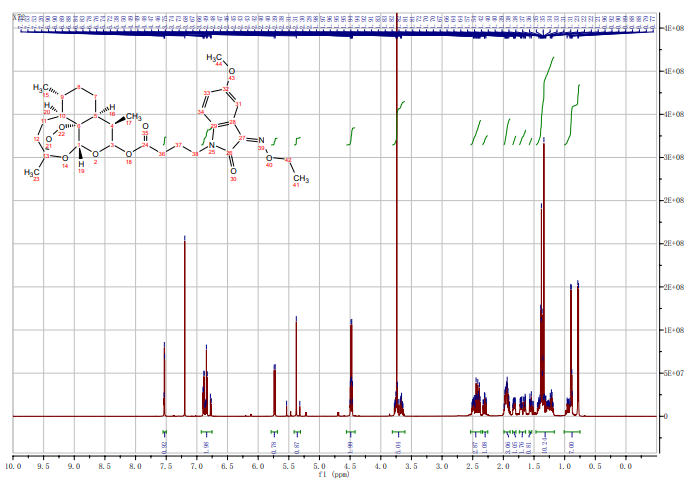


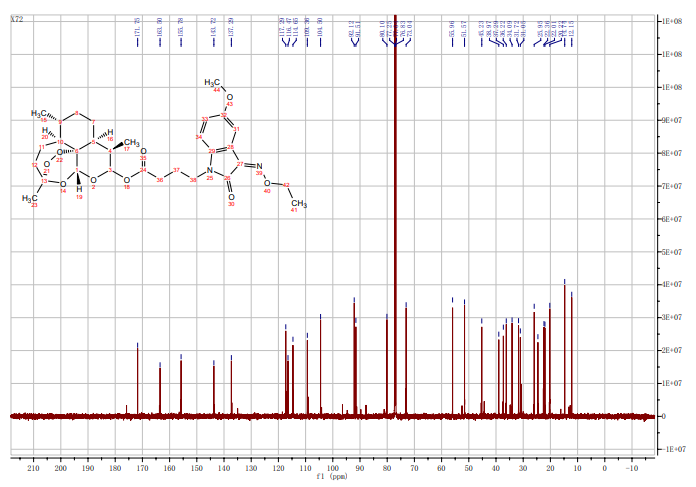


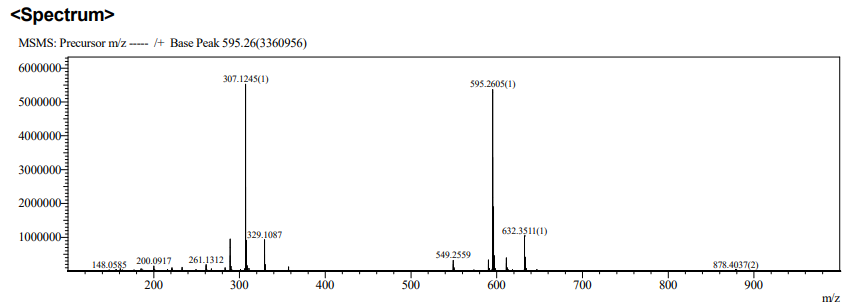


(3*R*,5a*S*,6*R*,8a*S*,9*R*,12*R*,12a*R*)-3,6,9-trimethyldecahydro-12*H*-3,12-epoxy[1,2]dioxepino[4,3-*i*]isochromen-10-yl 4-(3-((benzyloxy)imino)-5-methoxy-2-oxoindolin-1-yl)butanoate (**7u**)

^1^H NMR (600 MHz, CDCl_3_) δ 0.77-0.96 (m, 7H), 1.18-1.42 (m, 7H), 1.53-1.57 (m, 1H), 1.63-1.72 (m, 2H), 1.80-1.84 (m, 1H), 1.90-1.97 (m, 3H), 2.27-2.33 (m, 1H), 2.38-2.50 (m, 3H), 3.63-3.77 (m, 5H), 5.37 (s, 1H), 5.54 (s, 2H), 5.72 (d, *J* = 4.0 Hz, 1H), 6.75-6.89 (m, 2H), 7.27-7.38 (m, 5H), 7.49 (d, *J* = 2.0 Hz, 1H). ^13^C NMR (150 MHz, CDCl_3_) 171.74, 163.38, 156.79, 144.33, 137.41, 136.29, 128.60, 128.49, 128.44, 128.35, 128.33, 117.66, 116.38, 114.71, 109.48, 104.30, 92.14, 91.61, 80.10, 79.31, 55.87, 51.57, 45.23, 39.02, 37.29, 36.22, 34.09, 31.72, 31.04, 25.95, 24.89, 22.35, 22.01, 20.22, 12.15. HRMS-ESI: m/z Calcd for C_35_H_42_N_2_O_9_Na [M+Na]^+^: 657.2783; Found: 657.2741.


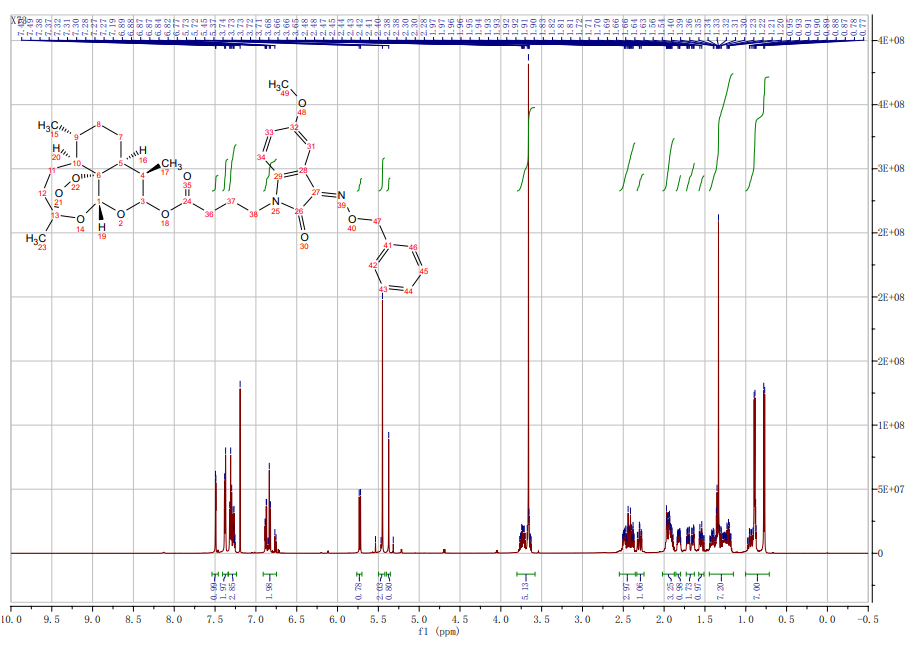


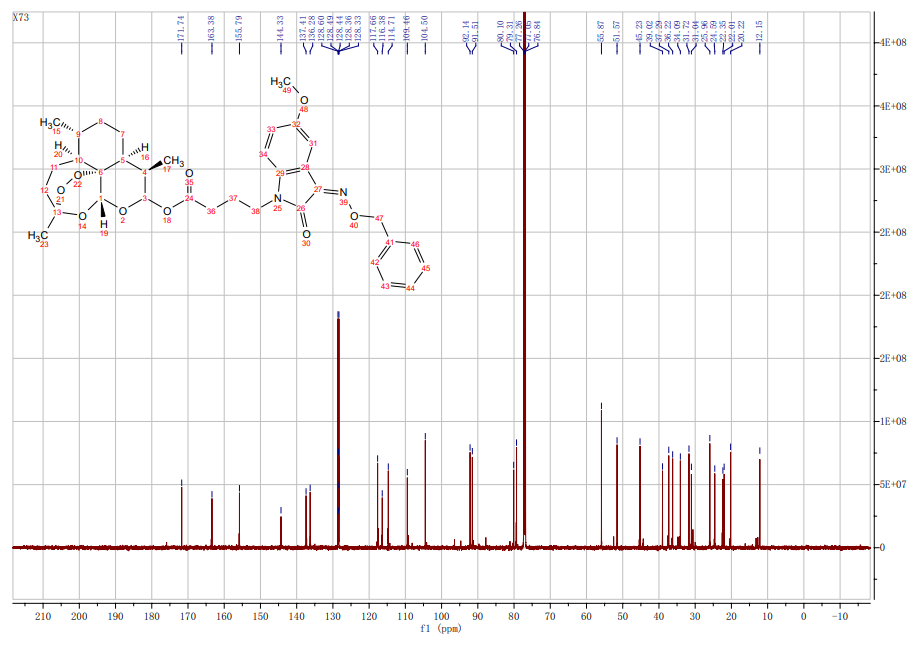


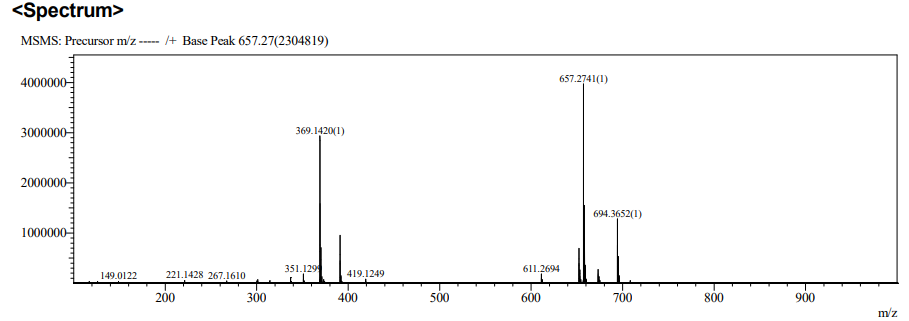


**Cell images**


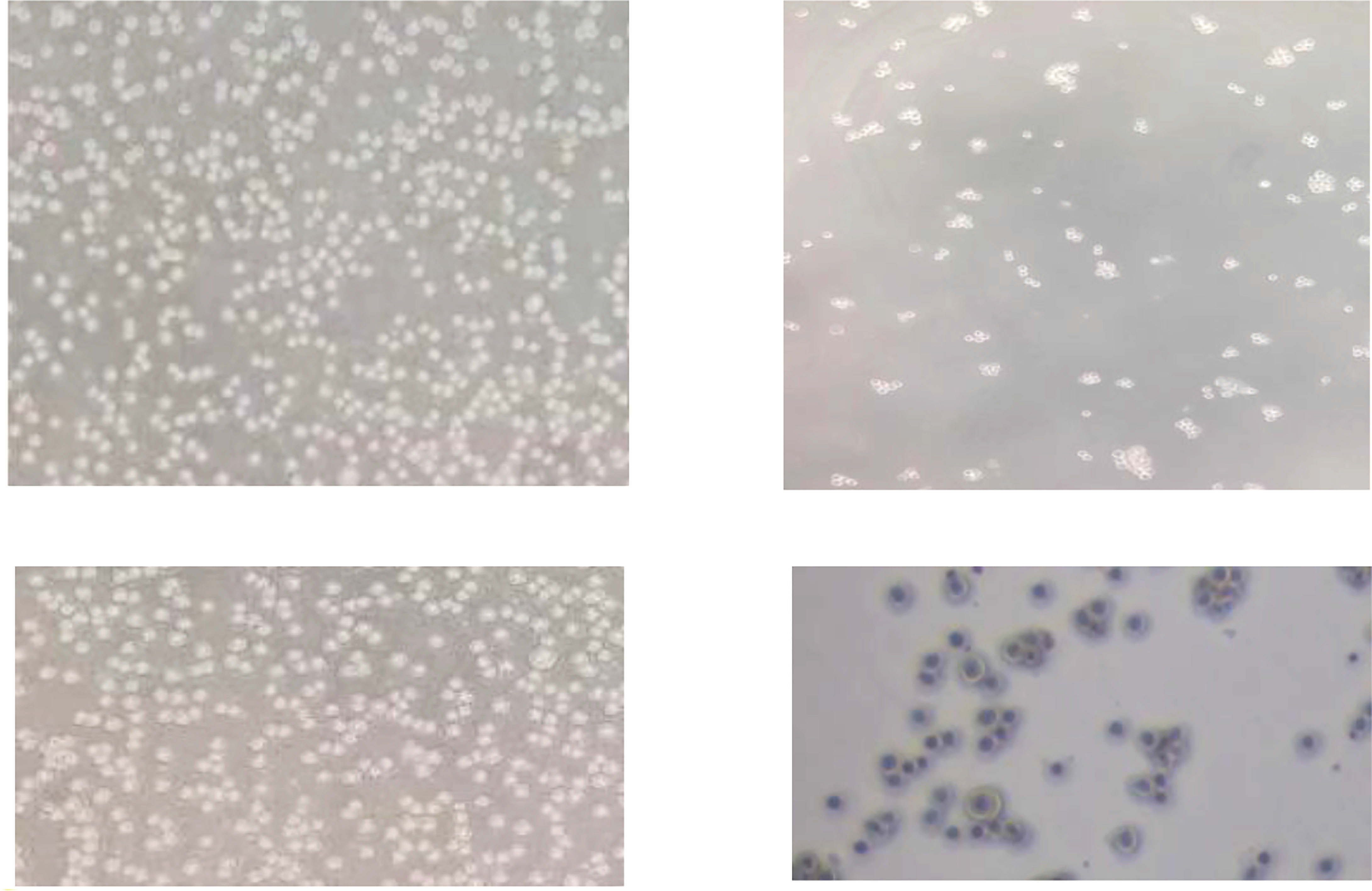


Cell images of CCRF-CEM and K562 cells before and after treatment of hybrid 7d. Cell image of CCRF-CEM cells (A) before treatment of hybrid 7d; (B) after treatment of hybrid 7d; Cell image of K562 cells (C) before treatment of hybrid 7d; (D) after treatment of hybrid 7d.
